# Dichloro-Phenyl-Benzotriazoles: A New Selective Class of Human Respiratory Syncytial Virus Entry Inhibitors

**DOI:** 10.3389/fchem.2019.00247

**Published:** 2019-04-16

**Authors:** Sandra Piras, Giuseppina Sanna, Antonio Carta, Paola Corona, Roberta Ibba, Roberta Loddo, Silvia Madeddu, Paola Caria, Suzana Aulic, Erik Laurini, Maurizio Fermeglia, Sabrina Pricl

**Affiliations:** ^1^Department of Chemistry and Pharmacy, University of Sassari, Sassari, Italy; ^2^Department of Biomedical Sciences, University of Cagliari, Cagliari, Italy; ^3^Molecular Biology and Nanotechnology Laboratory (MolBNL@Units), DEA, University of Trieste, Trieste, Italy

**Keywords:** dichloro-phenylbenzotriazoles, RSV strain B1 *cp-52*, fusion protein, plasmid-based reporter assay, *in silico* modeling

## Abstract

Human Respiratory Syncytial Virus (RSV) is the primary cause of bronchopneumonia in infants and children worldwide. Clinical studies have shown that early treatments of RSV patients with ribavirin improve prognosis, even if the use of this drug is limited due to myelosuppression and toxicity effects. Furthermore, effective vaccines to prevent RSV infection are currently unavailable. Thus, the development of highly effective and specific antiviral drugs for pre-exposure prophylaxis and/or treatment of RSV infections is a compelling need. In the quest of new RSV inhibitors, in this work we evaluated the antiviral activity of a series of variously substituted 5,6-dichloro-1-phenyl-1(2)H-benzo[d][1,2,3]triazole derivatives in cell-based assays. Several 1- and 2-phenyl-benzotriazoles resulted fairly potent (μM concentrations) inhibitors of RSV infection in plaque reduction assays, accompanied by low cytotoxicity in human highly dividing T lymphoid-derived cells and primary cell lines. Contextually, no inhibitory effects were observed against other RNA or DNA viruses assayed, suggesting specific activity against RSV. Further results revealed that the lead compound 10d was active during the early phase of the RSV infection cycle. To understand whether 10d interfered with virus attachment to target cells or virus-cell fusion events, inhibitory activity tests against the RSV mutant strain B1 cp-52—expressing only the F envelope glycoprotein—and a plasmid-based reporter assay that quantifies the bioactivity of viral entry were also performed. The overall biological results, in conjunction with *in silico* modeling studies, supported the conclusion that the RSV fusion process could be the target of this new series of compounds.

## Introduction

Viral pneumonia is an increasing health problem worldwide, and the incidence and number of viruses known to induce respiratory diseases have expanded in recent years (Lee and Qureshi, 2013). Human Respiratory Syncytial Virus (RSV) is the main etiological agent of pneumonia, bronchiolitis, and lower respiratory tract infections (LRTIs). In particular, RSV is the leading cause of acute LRTIs in children less than 5 years of age (Wang et al., 1995; Noyola et al., 2007), and the responsible for substantial disease burden in the elderly, similar to that of non-pandemic influenza A (Falsey et al., 2005; Falsey, 2007). Every year, this pathogen causes 30 million LRTIs and the annual death toll from RSV-related diseases is now ~ 200,000 (Nair et al., 2010).

RSV transmission occurs mainly through the eye and nose, rather than the mouth. This may be via large-particle aerosols or droplets, requiring close contact. The virus, however, does not seem capable of traversing distances by small-particle aerosols. Nevertheless, it is able to remain infectious on various environmental surfaces, suggesting fomites as a source of spread. These characteristics imply that RSV can be easily spread on hospital wards during a community break of RSV infection. The potential for nosocomial RSV infections is further enhanced by the crowding on pediatric wards that occur during such a community outbreak since, particularly in winter and early spring, a great proportion of admissions are infants with LRTI-related diseases who shed the virus in particularly high titer.

Actually, a prophylactic strategy based on a humanized neutralizing antibody against RSV is available to protect newborn babies at high-risk, such as preterm infants and those suffering from cardiovascular diseases and immunodeficiencies (Feltes et al., 2003; Cardenas et al., 2005). However, this approach is costly and unaffordable for most public health systems worldwide.

Likewise, aerosolized ribavirin (a synthetic guanosine analog) is available for treating RSV replication. The mechanism of action of this drug is based on the inhibition of RNA transcription; as such, ribavirin is characterized by a broad spectrum of antiviral activity, potential toxicity, besides relatively high cost (Ventre and Randolph, 2007). Moreover, the use of ribavirin is still limited because beneficial effects on clinical outcomes remain unproved (Ohmit et al., 1996; Snell, 2001). This scenario makes RSV—a negative-sense, single-stranded, enveloped RNA member of the *Pneumoviridae* family—an important target for the research and development of new antivirals.

The RSV RNA genome codes for key internal structural proteins (the matrix protein M and nucleoprotein N), proteins required for a functional polymerase complex (the phosphoprotein P and polymerase L), nonstructural proteins involved in evasion of the innate immune response (NS-1 and NS-2), externally exposed transmembrane glycoproteins (the small hydrophobic protein SH, th3 attachment protein G, and fusion protein F), and the regulatory M2 proteins (the M2-1 antitermination protein and M2-2, involved in transcription/replication regulation). RSV entry into target cells is mediated by the two glycoproteins G and F. These glycopolypeptides are packed in a dense layer on the viral surface, together with a third, small hydrophobic polypeptide (SH), whose function remains unknown. Primary adsorption of the virus to the target cell is generally promoted by the G protein, with sialic acid residues or cell surface proteins serving as receptors. Viral entry is then facilitated by the fusion F protein that adopts a metastable prefusion conformation when in its active form. After attachment of F to host-cell factors, one or more of these factors trigger the protein conformational rearrangement that results in fusion of the viral and cellular membranes. Since F is essential for RSV infection, humans elicit neutralizing antibodies that target it, with the most potent recognizing the prefusion conformation. Accordingly, this conformation of F is considered to be the ideal vaccine antigen, and antibodies and small molecules that disrupt its structure and function constitute a very active field of current research.

During the last two decades (starting by Sanna et al., 1992), our group has designed, synthesized and tested for biological activity a large number of 1(2)(3)*H*-benzo[d][1,2,3]triazole derivatives. The phenotypic screening approach to the biological evaluation against different pharmacological targets of 1(2)(3)H-benzo[d][1,2,3]triazole (hereafter named benzotriazole) derivatives (Briguglio et al., 2015) led to the discovery of compounds endowed with *in vitro* selective activity against ssRNA-compounds that exhibited specifically potent activity against RSV.

**Scheme 1:**
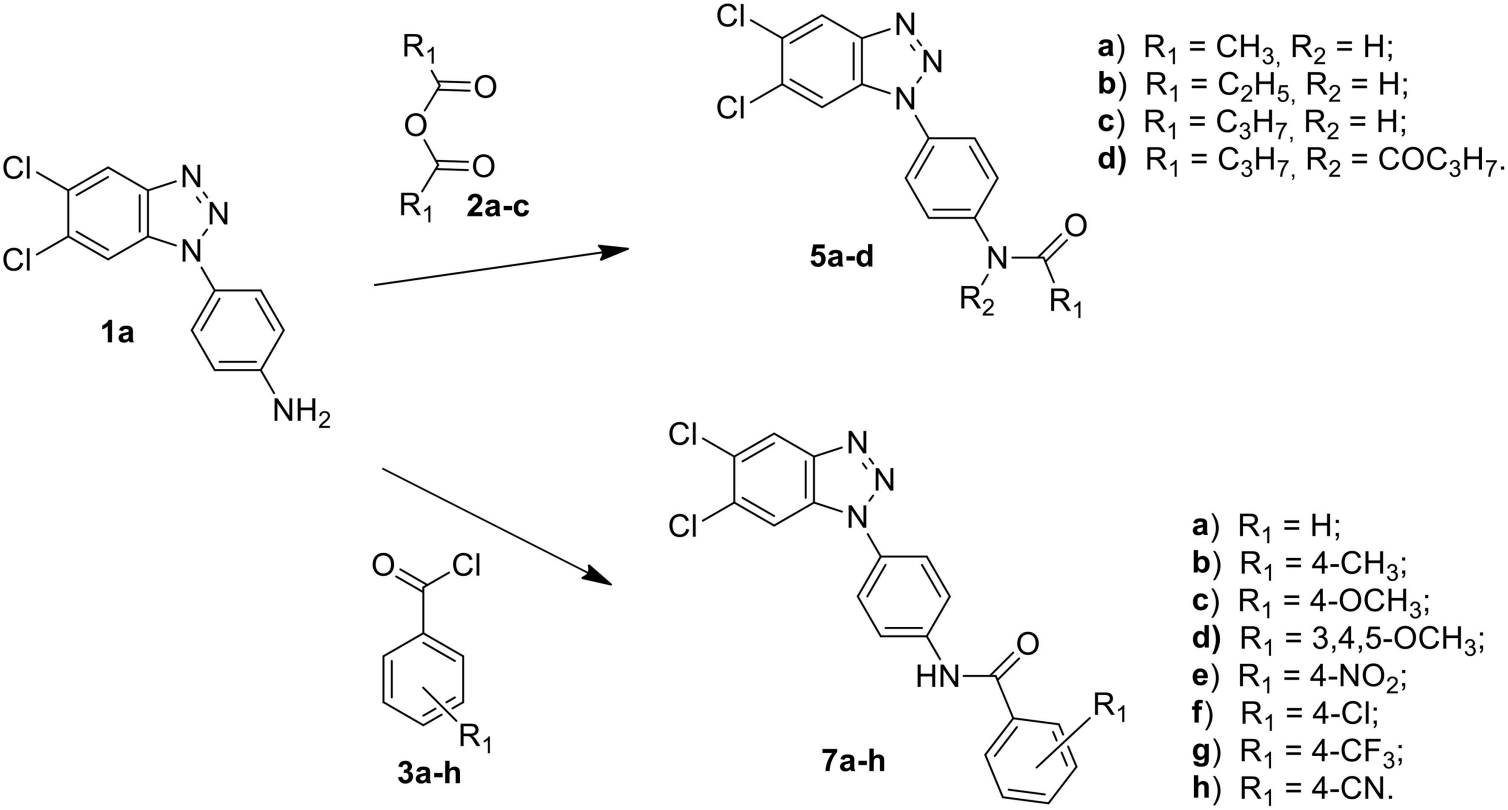
Synthesis of 5,6-dichloro-1-phenyl-benzotriazole amides **5a–d** and **7a–h**.

**Scheme 2:**
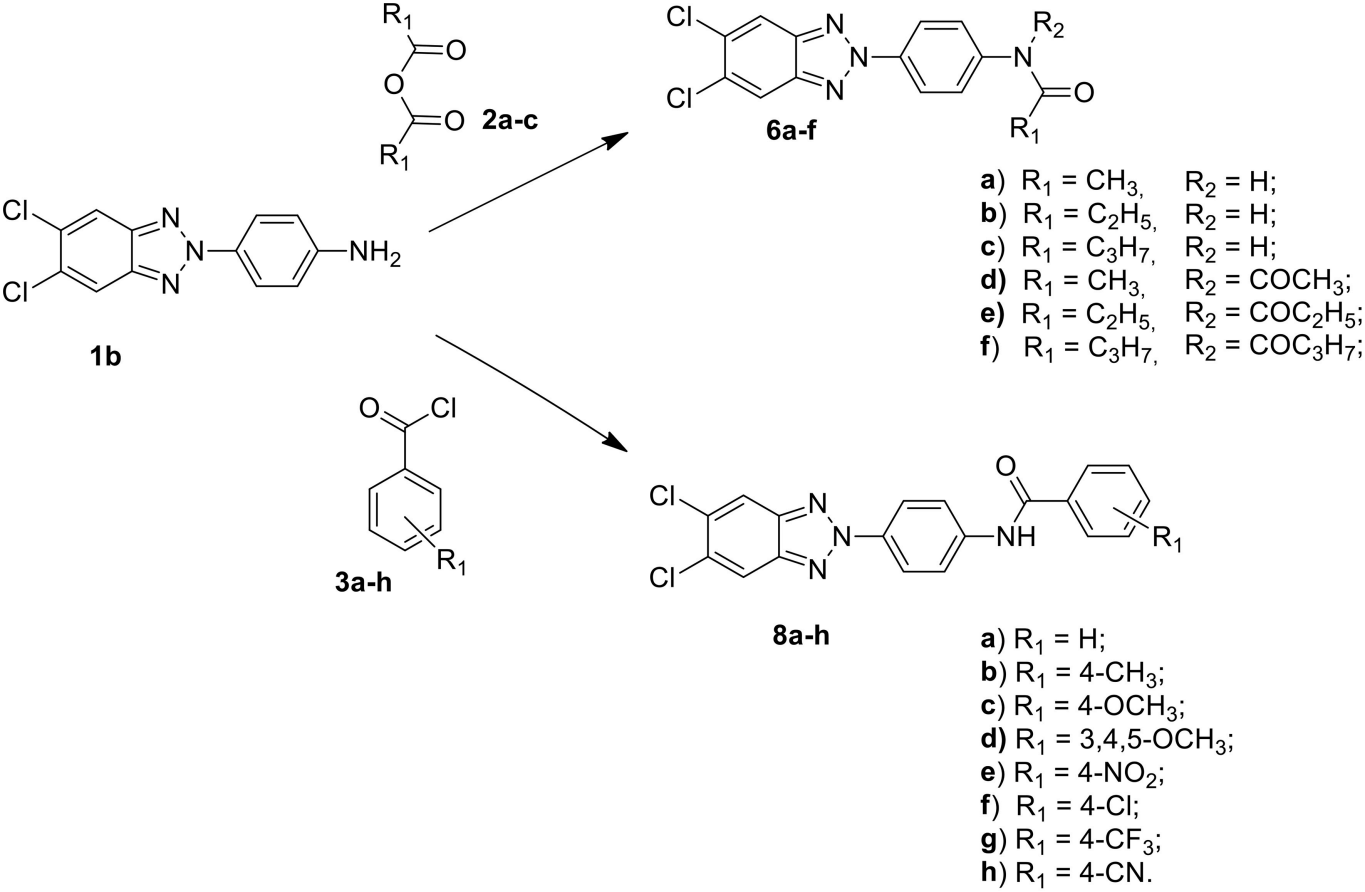
Synthesis of 5,6-dichloro-2-phenyl-benzotriazole amides **6a–f** and **8a–h**.

**Scheme 3:**
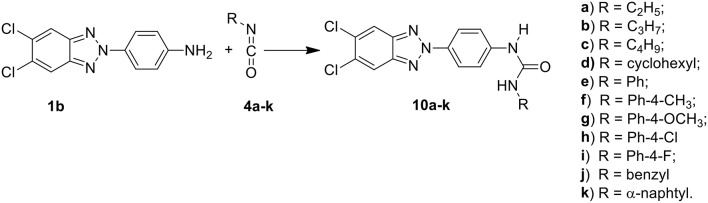
Synthesis of 5,6-dichloro-2-phenyl-benzotriazole urea derivatives **10a–k**.

Among these, the most potent ones carried a chlorine atom at position 5 of the heterocyclic scaffold. Given the beneficial role of the alogen substituent and with the aim of further potentiating the anti-RSV activity of benzotriazole compounds, in this paper we report the design and synthesis of 5,6-dichloro-1-phenyl-1H-benzotriazole amides (**5a-d** and **7a-h**) (Scheme 1) and 5,6-dichloro-2-phenyl-2H-benzotriazole derivatives **6a-f**, **8a-h** and **10a-k** (Schemes 2, 3) as potential RSV inhibitors.

The antiviral activity against RSV and the eventual related toxicity of these compounds in cell-based assays are next described, together with the results of an investigation aimed at understanding their mode of action in cell-, plasmid- and computer-based assays. Finally, results of the antiviral selectivity of these dichloro-phenyl-benzotriazoles, their cytotoxicity and antiviral activity against representative DNA, ± single-stranded RNA, and double-stranded genome (dsRNA) viruses families obtained in cell-based assays are also reported (see Supplementary Material).

## Materials and Methods

### General Experimental

Compound melting points (m.p.s) were taken in open capillaries in a Köfler hot stage or Digital Electrothermal melting point apparatus and are uncorrected. ^1^H Nuclear Magnetic Resonance (NMR) spectra were determined in CDCl_3_/DMSO, and were recorded with a Bruker Avance III 400 NanoBay (400 Mz) and a Varian XL-200 (200 MHz) instruments. Chemical shifts (™scale) are reported in parts per million (ppm) downfield from tetramethylsilane (TMS) used as internal standard. The chemical shift values are reported in ppm (δ) and coupling constants (*J*) in Hertz (Hz). Signal multiplicities are represented by: s (singlet), d (doublet), dd (double doublet), t (triplet), q (quadruplet) and m (multiplet). The assignment of exchangeable protons (O*H* and N*H*) was confirmed by the addition of D_2_O. ^13^C NMR were determined in DMSO and were recorded at 400 MHz with Bruker Avance III 400 NanoBay. Mass spectra (MS) were performed on combined HP 5790-HP 5970 GC/MS apparatus or with a combined Liquid Chromatograph-Agilent 1100 series Mass Selective Detector (MSD). Column chromatography was performed using 70–230 mesh (Merck silica gel 60). Light petroleum refers to the fraction with b.p. 40–60°C. The progress of the reactions, the retardation factor (R_f_) and the purity of the final compounds were monitored by TLC using Merck F-254 commercial plates. Analyses indicated by the symbols of the elements were within ± 0.4% of the theoretical values. Acetic anhydride (**2a**), propionic anhydride (**2b**) and butyric anhydride (**2c**), 4-R benzoyl chloride (**3a-h**), alkyl- and aryl-isocyanates (**4a**-**k**) were commercially available (from Sigma Aldrich, USA), 1-(4-aminophenyl)-5,6-dichlorobenzotriazole (**1a)** and 2-(4-aminophenyl)-5,6-dichlorobenzotriazole **(1b**) were prepared following the procedure previously described by us (Carta et al., 2006).

### General Procedure for Preparation of N-[4-(1*H*(2*H*)-benzo[*d*][1,2,3]triazol-1(2)-yl)phenyl] alkylcarboxamides (5a-d and 6a-f)

A stirred suspension of the 1-(4-aminophenyl)-5,6-dichlorobenzotriazole (**1a)** or 2-(4-aminophenyl)-5,6-dichlorobenzotriazole **(1b**) (1.4 mmol) in the appropriate anhydride (acetic, propionic and butyric anhydride) (4 mL), was heated to 100°C for 1h. After cooling to r.t. and dilution with an equal volume of water (4 mL), the resulting precipitate was filtered off, washed with water and dried. The afforded crude products were purified by flash chromatography using as eluent a mixture of petroleum ether/ethyl acetate in ratio 7:3. Besides the desired compounds, the diacylated derivatives **5d** and **6d-f** were also collected. All solid compounds were crystallized from ethanol. Melting points, yields, analytical and spectroscopy data are reported below.

### General Procedure for Preparation of N-[4-(1*H*(2*H*)-benzo[*d*][1,2,3]triazol-1(2)-yl)phenyl]p-R-arylcarboxamides (7a-h and 8a-h)

To a stirred solution of the 1-(4-aminophenyl)-5,6-dichlorobenzotriazole (**1a)** and 2-(4-aminophenyl)-5,6-dichlorobenzotriazole **(1b**) (1.4 mmol) in anhydrous dimethylformamide (DMFa) (10 mL) was added dropwise a solution in DMFa (2 mL) of appropriate R-benzoyl chloride (1.68 mmol) (ratio 1: 1+20%). The solution was then stirred at 80°C for further 0.5 h (**7a**,**d** and **8d**,**f**), 1h (**7b**, **c**, **e, g** and **8a**), 1.5 h (**8b**), 2h (**7f**), 3 h (**8e**), 12 h (**8c**), 2 4h (**7h**), and 72 h (**7g**). After cooling, the obtained solids were filtered, and water was added to achieve quantitative precipitation. The resulting precipitates were collected by filtration, washed with water, and the crude products were purified by flash chromatography (**7c**, C/M = 95/5), (**7d**, C/M = 98/2), (**7g**, **7h**, PE/EA = 7/3), (**8b**, C/M = 9/1), (**8c**, PE/DE = 3/7), (**8d**, PE/EA = 6/4), **(8f**, PE/DE = 75/25) or via recrystallization by ethanol (**7a**, **7b**, **7e**, **7f**, **8a**, **8e**, **8g**, **8h**). C: chloroform, M: methanol, PE: petroleum ether, DE: diethyl ether, EA: ethyl acetate.

### General Procedure for Preparation of 1-[4-(5,6-dichloro-2*H*-benzo[*d*][1,2,3]triazol-2-yl)phenyl]-3-alkyl and aryl-uree (10a-k)

To a stirred solution of the 2-(4-aminophenyl)-5,6-dichlorobenzotriazole **(1b**) (1.26 mmol) in anhydrous N,N-dimethylformamide (DMFa) (6 mL), 3.78 mmol (ratio 1:3) of the required isocyanate (**4a-k**) were added in anhydrous N,N-dimethylformamide. The mixture was stirred at 80°C for 24 h (**10c**), 44 h (**10b**), 3 days (**10a,k**), 110°C for 24 h (**10f,g,i,l**), 48 h (**10h**), and 72 h (**10e**). The reaction mixture was cooled to room temperature and the precipitates of **4a-k** were directly collected by filtration. For all other compounds, the liquor mothers were evaporated to dryness and the crude products were triturated with ether to obtain the corresponding solids, which were purified either by recrystallization with ethanol (**10a,c,d,f-l**) or by flash chromatography using a mixture of petroleum ethyl ether/diethyl acetate 6/4 (**10b,e,k**).

### *N*-[4-(5,6-dichloro-1*H*-benzo[*d*][1,2,3]triazol-1-yl)phenyl]acetamide (5a)

C_14_H_10_Cl_2_N_4_O MW 321.16 Elem. Anal. Calc. C 52.36, H 3.14, N 17.45. Found C 52.70, H 3.09, N 17.35. Compound total yield 82%; m.p. 209–211°C; TLC (diethylether): R_f_ 0.23. IR (nujol) ν: 1,700, 3,400 cm^−1^. UV (EtOH) λ_max_ 245 nm. δ 10.24 (s, 1H, NH), 8.29 (s, 1H, H-4), 7.95 (s, 1H, H-7), 7.89 (d, *J* = 8.6 Hz, 2H, H-2′, 6′), 7.45 (d, *J* = 8.6 Hz, 2H, H-3′, 5′) 2.38 (s, 3H, CH_3_). ^13^C-NMR (DMSO): 172.4 (CO), 144.6 (C), 140.2 (C), 131.7 (C), 131.0 (C), 130.3 (C), 127.6 (C), 123.6 (2CH), 121.2 (CH), 120.8 (2CH), 112.8 (CH), 23.4 (CH_3_). GC/MS: m/z 321, 323 [M+].

### *N*-[4-(5,6-dichloro-1*H*-benzo[*d*][1,2,3]triazol-1-yl)phenyl]propionamide (5b)

C_15_H_12_Cl_2_N_4_O MW 335.19 Elem. Anal. Calc. C 53.75, H 3.61, N 16.72. Found C 53.60, H 3.41, N 16.68. Compound total yield 33%; m.p. 175–177°C; TLC (petroleum ether/ethyl acetate 7/3): R_f_ 0.36. IR (nujol) ν: 1700, 3380 cm^−1^. UV (EtOH) λ_max_ 217, 260 nm. ^1^HNMR (CDCl_3_ /DMSO): δ 10.24 (s, 1H, NH), 8.25 (s, 1H, H-4), 8.06 (s, 1H, H-7), 7.83 (d, *J* = 8.6 Hz, 2H, H-2′, 6′), 7.67 (d, *J* = 8.6 Hz, 2H, H-3′, 5′), 2.46 (q, *J* = 7.2 Hz, 2H, CH_2_), 1.28 (t, *J* = 7.2 Hz, 3H, CH_3_). ^13^C-NMR (DMSO): 172.4 (CO), 144.6 (C), 140.2 (C), 131.8 (C), 131.1 (C), 130.3 (C), 127.6 (C), 123.6 (2CH), 121.1 (CH), 120.8 (2CH), 112.8 (CH), 29.6 (CH_2_), 9.5 (CH_3_). GC/MS: m/z 334, 336 [M+].

### *N*-[4-(5,6-dichloro-1*H*-benzo[*d*][1,2,3]triazol-1-yl)phenyl]butanamide (5c)

C_16_H_14_Cl_2_N_4_O MW 349.21 Elem. Anal. Calc. C 55.03, H 4.04, N 16.04. Found C 55.33 H 4.28 N 15.84. Compound total yield 30%; m.p. 202–204°C; TLC (petroleum ether/ethyl acetate 7/3): R_f_ 0.73. IR (nujol) ν: 1670, 3390 cm^−1^. UV (EtOH) λ_max_ 217, 262 nm. ^1^HNMR (CDCl_3_ /DMSO): δ 10.30 (s, 1H, NH), 8.25 (s, 1H, H-4), 7.84 (s, 1H, H-7), 7.82 (d, *J* = 8.8 Hz, 2H, H-2′, 6′), 7.67 (d, *J* = 8.8 Hz, 2H, H-3′, 5′), 2.41 (q, *J* = 7.2 Hz, 2H, CH_2_), 1.81 (q, *J* = 7.4 Hz, 2H, CH_2_), 1.05 (t, *J* = 7.2 Hz, 3H, CH_3_). ^13^C-NMR (DMSO): 171.7 (CO), 144.6 (C), 140.1 (C), 137.9 (C), 131.8 (C), 131.4 (C), 130.3 (C), 127.6 (C), 123.6 (2CH), 120.9 (CH), 119.9 (2CH), 112.8 (CH), 37.9 (CH_2_), 18.5 (CH_2_), 13.6 (CH_3_). GC/MS: m/z 348, 350 [M+].

### N-butyryl*-N*-[4-(5,6-dichloro-1*H*-benzo[*d*][1,2,3]triazol-1-yl)phenyl] butyramide (5d)

C_20_H_20_Cl_2_N_4_O_2_ MW 419.09 Elem. Anal. Calc. C 57.29, H 4.81, N 13.36. Found C 57.33, H 4.58, N 13.84. Compound total yield 10%; m.p. 202–204°C; TLC (petroleum ether/ethyl acetate 7/3): R_f_ 0.74. IR (nujol) ν: 1,670 cm^−1^. UV (EtOH) λ_max_ 217, 262 nm. ^1^HNMR (CDCl_3_ /DMSO): δ 8.28 (s, 1H, H-4), 7.90 (s, 1H, H-7), 7.78 (d, *J* = 8.8 Hz, 4H, H-2′, 3′, 5′, 6′), 2.52 (t, *J* = 7.2 Hz, 2H, CH_2_CO), 2.38 (t, *J* = 7.2 Hz, 2H, CH_2_CO), 1.82 (q, *J* = 7.2 Hz, 2H, CH_2_), 1.74 (q, *J* = 7.2 Hz, 2H, CH_2_), 1.04 (t, *J* = 7.2 Hz, 3H, CH_3_), 0.99 (t, *J* = 7.2 Hz, 3H, CH_3_). ^13^C-NMR (DMSO): 171.5 (CO), 144.6 (C), 140.1 (C), 137.9 (C), 131.8 (C), 131.4 (C), 130.3 (C), 127.6 (C), 123.6 (2CH), 120.9 (CH), 119.9 (2CH), 112.8 (CH), 37.9 (2CH_2_), 18.5 (2CH_2_), 13.6 (2CH_3_). GC/MS: m/z 418, 420 [M+].

### *N*-[4-(5,6-dichloro-2*H*-benzo[*d*][1,2,3]triazol-2-yl)phenyl]acetamide (6a)

C_14_H_10_Cl_2_N_4_O MW 321.16 Elem. Anal. Calc. C 52.36, H 3.14, N 17.45. Found C 52.02, H 3.37, N 17.70. Compound total yield 74%; m.p. > 290°C; TLC (diethylether): R_f_ 0.30. IR (nujol) ν: 1650, 3370 cm^−1^. UV (EtOH) λ_max_ 214, 332 nm. ^1^HNMR (CDCl_3_/DMSO): δ 10.32 (s, 1H, NH), 8.47 (s, 2H, H-4, 7), 8.23 (d, *J* = 8.4 Hz, 2H, H-2′, 6′), 7.86 (d, *J* = 8.4 Hz, 2H, H-3′, 5′), 2.11 (s, 3H, CH_3_). ^13^C-NMR (DMSO): 168.8 (CO), 143.2 (2C), 140.9 (C), 134.0 (C), 130.4 (2C), 121.1 (2CH), 119.6 (2CH), 119.4 (2CH), 24.1 (CH_3_). GC/MS: m/z 320, 322 [M+].

### *N*-[4-(5,6-dichloro-2*H*-benzo[*d*][1,2,3]triazol-2-yl)phenyl]propanamide (6b)

C_15_H_12_Cl_2_N_4_O MW 335.19 Elem. Anal. Calc. C 53.75, H 3.61, N 16.72. Found C 53.47, H 3.93, N 17.00. Compound total yield 35%; m.p. 258–260°C; TLC (petroleum ether/ethyl acetate 7/3): R_f_ 0.73. IR (nujol) ν: 1655, 3380 cm^−1^. UV (EtOH) λ_max_ 217, 342 nm. ^1^HNMR (CDCl_3_ /DMSO): δ 10.20 (s, 1H, NH), 8.37 (s, 2H, H-4, 7), 8.22 (d, *J* = 9.0 Hz, 2H, H-2′, 6′), 7.88 (d, *J* = 9.0 Hz, 2H, H-3′, 5′), 2.38 (q, *J* = 7.4 Hz, 2H, CH_2_), 1.13 (t, *J* = 7.4 Hz, 3H, CH_3_). ^13^C-NMR (DMSO): 172.4 (CO), 143.2 (2C), 140.9 (C), 134.0 (C), 130.4 (2C), 121.1 (2CH), 119.6 (2CH), 119.4 (2CH), 29.6 (CH_2_), 9.5 (CH_3_). GC/MS: m/z 334, 336 [M+].

### *N*-[4-(5,6-dichloro-2*H*-benzo[*d*][1,2,3]triazol-2-yl)phenyl]butanamide (6c)

C_16_H_14_Cl_2_N_4_O MW 349.21 Elem. Anal. Calc. C 55.03, H 4.04 N, 16.04. Found C 55.40, H 4.36, N 15.77. Compound total yield 72%; m.p. 243–245°C; TLC (petroleum ether/ethyl acetate 7/3): R_f_ 0.64. IR (nujol) ν: 1656, 3330 cm^−1^. UV (EtOH) λ_max_: 219, 330 nm. ^1^HNMR (CDCl_3_ /DMSO): δ 10.41 (s, 1H, NH), 8.46 (s, 2H, H-4, 7), 8.22 (d, *J* = 8.4 Hz, 2H, H-2′, 6′), 7.91 (d, *J* = 8.4 Hz, 2H, H-3′, 5′), 2.36 (t, *J* = 7.4 Hz, 2H, CH_2_CO), 1.64 (q, *J* = 7.4 Hz, 2H, CH_2_CH_3_), 0.94 (t, *J* = 7.4 Hz, 3H, CH_3_). ^13^C-NMR (DMSO): 171.6 (CO), 144.1 (2C), 141.0 (C), 133.9 (C), 130.3 (2C), 121.0 (2CH), 119.6 (2CH), 119.4 (2CH), 38.3 (CH_2_), 18.5 (CH_2_), 13.6 (CH_3_). GC/MS: m/z 348, 350 [M+].

### *N-acetyl-N*-[4-(5,6-dichloro-2*H*-benzo[*d*][1,2,3]triazol-2-yl)phenyl]acetamide (6d)

C_16_H_12_Cl_2_N_4_O_2_ MW 363.20 Elem. Anal. Calc. C 52.91, H 3.33, N 15.43. Found C 53.20, H 3.66, N 15.77. Compound total yield 15%; m.p. 224–226°C; TLC (petroleum ether/ethyl acetate 7/3): R_f_ 0.64. IR (nujol) ν: 1,660, cm^−1^. UV (EtOH) λ_max_ 220, 330 nm. ^1^HNMR (CDCl_3_ /DMSO): δ 8.61 (s, 2H, H-4, 7), 8.39 (d, *J* = 8.0 Hz, 2H, H-2′, 6′), 7.93 (d, *J* = 8.0 Hz, 2H, H-3′, 5′), 2.42 (s, 3H, CH_3_), 2.22 (s, 3H, CH_3_). ^13^C-NMR (DMSO): 169.8 (2CO), 148.7 (2C), 145.4 (2C), 136.1 (2C), 126.4 (2CH), 124.9 (4CH), 26.8 (CH_3_), 23.4 (CH_3_). GC/MS: m/z 362, 364 [M+].

### N-propionyl*-N*-[4-(5,6-dichloro-2*H*-benzo[*d*][1,2,3]triazol-2-yl)phenyl] propionamide (6e)

C_18_H_16_Cl_2_N_4_O_2_ MW 391.25 Elem. Anal. Calc. C 55.26, H 4.12, N 14.32. Found C 55.47, H 4.43, N 13.90. Compound total yield 15%; m.p. 238–240°C; TLC (petroleum ether/ethyl acetate 7/3): R_f_ 0.73. IR (nujol) ν: 1655 cm^−1^. UV (EtOH) λ_max_ 218, 340 nm. ^1^HNMR (CDCl_3_ /DMSO): δ 8.60 (s, 2H, H-4, 7), 8.42 (d, *J* = 9.0 Hz, 2H, H-2′, 6′), 7.88 (d, *J* = 9.0 Hz, 2H, H-3′, 5′), 2.71 (q, *J* = 7.4 Hz, 2H, CH_2_), 2.59 (q, *J* = 7.2 Hz, 2H, CH_2_), 1.27 (t, *J* = 7.4 Hz, 3H, CH_3_), 1.21 (t, *J* = 7.4 Hz, 3H, CH_3_). ^13^C-NMR (DMSO): 171.0 (2CO), 143.2 (2C), 140.9 (C), 134.0 (C), 130.4 (2C), 121.1 (2CH), 119.4 (4CH), 29.6 (CH_2_), 29.2 (CH_2_), 9.5 (2CH_3_). GC/MS: m/z 390, 392 [M+].

### N-butyryl*-N*-[4-(5,6-dichloro-2*H*-benzo[*d*][1,2,3]triazol-2-yl)phenyl] butyramide (6f)

C_20_H_20_Cl_2_N_4_O_2_ MW 419.30 Elem. Anal. Calc. C 57.29, H 4.81, N 13.36. Found C 57.40, H 4.46, N 13.77. Compound total yield 20%; m.p. 130–133°C; TLC (diethyl ether/petroleum ether 7/3): R_f_ 0.88. IR (nujol) ν: 1,684 cm^−1^. UV (EtOH) λ_max_ 224, 330 nm. ^1^HNMR (CDCl_3_ /DMSO): δ 8.38 (d, *J* = 9.0 Hz, 2H, H-2′, 6′), 8.08 (s, 2H, H-4, 7), 7.67 (d, *J* = 9.0 Hz, 2H, H-3′, 5′), 2.50 (t, *J* = 7.2 Hz, 2H, CH_2_CO), 2.34 (t, *J* = 7.2 Hz, 2H, CH_2_CO), 1.77 (q, *J* = 7.2 Hz, 2H, CH_2_), 1.35 (q, *J* = 7.2 Hz, 2H, CH_2_), 1.03 (t, *J* = 7.2 Hz, 3H, CH_3_), 0.95 (t, *J* = 7.2 Hz, 3H, CH_3_). ^13^C-NMR (DMSO): 171. (2CO), 143.5 (2C), 140.3 (2C), 130.9 (2C), 121.2 (2CH), 119.7 (4CH), 38.9 (CH_2_), 32.6 (2CH_2_), 17.6 (CH_2_), 13.4 (CH_3_). 13.2 (CH_3_). GC/MS: m/z 418, 420 [M+].

### *N*-[4-(5,6-dichloro-1*H*-benzo[*d*][1,2,3]triazol-1-yl)phenil]benzamide (7a)

C_19_H_12_Cl_2_N_4_O MW 383.23 Elem. Anal. Calc. C 59.55, H 3.16, N 14.62. Found C 60.20, H 3.16, N 14.92. Compound total yield 36%; m.p. 250–252°C; TLC (petroleum ether/ethyl acetate 7/3): R_f_ 0.55. IR (nujol) ν: 1648, 3370 cm^−1^. UV (EtOH) λ_max_ 216, 284 nm. ^1^HNMR (CDCl_3_ /DMSO): δ 9.98 (s, 1H, NH), 8.26 (s, 1H, H-4), 8.13 (d, *J* = 8.6 Hz, 2H, H-2′, 6′), 7.99-8.05 (m, 2H, Ph), 7.90 (s, 1H, H-7), 7.69 (d, *J* = 8.6 Hz, 2H, H-3′, 5′), 7.52-7.58 (m, 3H, Ph). ^13^C-NMR (DMSO): 165.9 (CO), 144.6 (C), 140.0 (C), 134.6 (C), 131.9 (CH), 131.8 (C), 131.1 (C), 130.9 (C), 128.4 (2CH), 127.7 (2CH), 127.6 (C), 124.2 (2CH), 121.2 (2CH), 120.9 (CH), 112.9 (CH). GC/MS: m/z 382, 384 [M+].

### *N*-[4-(5,6-dichloro-1*H*-benzo[*d*][1,2,3]triazol-1-yl)phenyl]-4-methylbenzamide (7b)

C_20_H_14_Cl_2_N_4_O MW 397.26 Elem. Anal. Calc. C 60.47, H 3.55, N 14.10. Found C 60.07, H 3.80, N 13.90. Compound total yield 24%; m.p. 226–228°C; TLC (petroleum ether/ethyl acetate 7/3): R_f_ 0.51. IR (nujol) ν: 1,680, 3,380 cm^−1^. UV (EtOH) λ_max_ 208, 282 nm. ^1^HNMR (CDCl_3_ /DMSO): δ 10.32 (s, 1H, NH), 8.29 (s, 1H, H-4), 8.16 (d, *J* = 8.6 Hz, 2H, H-2′, 6′), 7.93(d, *J* = 8.2 Hz, 2H, H-2′′, 6′′), 7.99 (s, 1H, H-7), 7.71 (d, *J* = 8.6 Hz, 2H, H-3′, 5′), 7.32 (d, *J* = 8.2 Hz, 2H, H-3′′, 5′′), 2.45 (s, 3H, CH_3_). ^13^C-NMR (DMSO): 165.6 (CO), 144.6 (C), 141.9 (C), 140.1 (C), 131.8 (C), 131.7 (C), 131.2 (C), 130.8 (C), 128.9 (2CH), 127.8 (2CH), 127.7 (C), 123.4 (2CH), 121.2 (2CH), 120.9 (CH), 112.9 (CH), 21.0 (CH_3_). GC/MS: m/z 396, 398 [M+].

### *N*-[4-(5,6-dichloro-1*H*-benzo[*d*][1,2,3]triazol-1-yl)phenyl]-4-methoxybenzamide (7c)

C_20_H_14_Cl_2_N_4_O_2_ MW 413.26 Elem. Anal. Calc. C 58.13, H 3.41, N 13.56. Found C 58.13, H 3.40, N 13.36. Compound total yield 54%; m.p. 193–195°C; TLC (petroleum ether/ethyl acetate 7/3): R_f_ 0.67. IR (nujol) ν: 1670, 3,360 cm^−1^. UV (EtOH) λ_max_ 210, 284 nm. ^1^HNMR (CDCl_3_ /DMSO): δ 10.23 (s, 1H, NH), 8.28 (s, 1H, H-4), 8.16 (d, *J* = 8.8 Hz, 2H, H-2′, 6′), 8.02 (d, *J* = 8.6 Hz, 2H, H-2′, 6′), 7.98 (s, 1H, H-7), 7.76 (d, *J* = 8.8 Hz, 2H, H-3′, 5′), 7.00 (d, *J* = 8.6 Hz, 2H, H-3′, 5′), 3.89 (s, 3H, OCH_3_). ^13^C-NMR (DMSO): 165.6 (CO), 162.6 (C), 141.9 (C), 140.1 (C), 131.8 (C), 131.7 (C), 131.2 (C), 130.8 (C), 128.9 (2CH), 127.8 (2CH), 127.7 (C), 123.4 (2CH), 121.2 (2CH), 120.9 (CH), 112.9 (CH), 55.4 (OCH_3_). GC/MS: m/z 412, 414 [M+].

### *N*-[4-(5,6-dichloro-1*H*-benzo[*d*][1,2,3]triazol-1-yl)phenyl]-3,4,5-trimethoxybenzamide (7d)

C_22_H_18_Cl_2_N_4_O_4_ MW 473.31 Elem Anal. Calc. C 55.83, H 3.83, N 11.84. Found C 56.03, H 4.00, N 12.00. This compound was obtained in 34% of total yield; m.p. 212–213°C; TLC (petroleum ether/ethyl acetate 7/3): R_f_ 0.84. IR (nujol) ν: 1,680, 3,390 cm^−1^. UV (EtOH) λ_max_ 197, 264, 318 nm. ^1^HNMR (CDCl_3_ /DMSO): δ 11.83 (s, 1H, NH), 8.27 (s, 1H, H-4), 7.95 (d, *J* = 8.8 Hz, 2H, H-2′, 6′), 7.89 (s, 1H, H-7), 7.74 (d, *J* = 8.8 Hz, 2H, H-3′, 5′),7.14 (s, 2H, H-2′′, 6′′), 3.97 (s, 9H, 3OCH_3_). ^13^C-NMR (DMSO): 165.6 (CO), 152.6 (2C-O), 141.9 (C-O), 140.1 (C), 131.8 (2C), 131.7 (C), 131.2 (C), 130.8 (2C), 128.9 (2CH), 123.4 (2CH), 121.2 (2CH), 120.9 (CH), 112.9 (CH), 60.1 (OCH_3_), 56.1 (2OCH_3_). GC/MS: m/z 472, 474 [M+].

### *N*-[4-(5,6-dichloro-1*H*-benzo[*d*][1,2,3]triazol-1-yl)phenyl]-4-nitrobenzamide (7e)

C_19_H_11_Cl_2_N_5_O_3_ MW 428.23 Elem. Anal. Calc. C 53.29, H 2.59, N 16.35. Found C 53.62, H 2.79, N 16.00. Compound total yield 15%; m.p. 238–240°C; TLC (petroleum ether/ethyl acetate 7/3): R_f_ 0.60. IR (nujol) ν: 1684, 3380 cm^−1^. UV (EtOH) λ_max_ 208 nm. ^1^HNMR (CDCl_3_ /DMSO): δ 10.81 (s, 1H, NH), 8.47 (s, 1H, H-4), 8.40 (d, *J* = 9.0 Hz, 2H, H-2′, 6′), 8.21 (d, *J* = 9.2 Hz, 2H, H-3′′, 5′′), 8.29 (s, 1H, H-7), 8.15 (d, *J* = 9.2 Hz, 2H, H-2′′, 6′′), 7.87 (d, *J* = 9.0 Hz, 2H, H-3′, 5′). ^13^C-NMR (DMSO): 166.9 (CO), 149.3 (C), 145.6 (C), 140.1 (C), 131.8 (C), 131.7 (C), 131.2 (C), 130.8 (C), 128.9 (2CH), 127.8 (2CH), 127.7 (C), 123.4 (2CH), 121.2 (2CH), 120.9 (CH), 112.9 (CH). GC/MS: m/z 427, 429 [M+].

### *N*-[4-(5,6-dichloro-1*H*-benzo[*d*][1,2,3]triazol-1-yl)phenyl] 4-chlorobenzamide (7f)

C_19_H_11_Cl_3_N_4_O MW 417.68 Elem. Anal. Calc. C 54.64, H 2.65, N 13.41. Found C 54.26, H 2.95, N 13.61. Compound total yield 48%; m.p. 234–236°C; TLC (petroleum ether/ethyl acetate 7/3): R_f_ 0.46. IR (nujol) ν: 1,650, 3,400 cm^−1^. UV (EtOH) λ_max_ 208, 234, 276 nm. ^1^HNMR (CDCl_3_ /DMSO): δ 10.27 (s, 1H, NH), 8.26 (s, 1H, H-4), 8.40 (d, *J* = 8.8 Hz, 2H, H-2′, 6′), 8.02 (d, *J* = 8.6 Hz, 2H, H-3′′, 5′′), 8.92 (s, 1H, H-7), 7.70 (d, *J* = 8.6 Hz, 2H, H-2′′, 6′′), 7.51 (d, *J* = 8.8 Hz, 2H, H-3′, 5′). ^13^C-NMR (DMSO): 164.7 (CO), 144.7 (C), 139.9 (C), 136.6 (C), 133.3 (C), 131.8 (C), 131.1 (C), 129.8 (2CH), 128.5 (2CH), 127.7 (2C), 123.4 (2CH), 121.3 (2CH), 121.0 (CH), 112.9 (CH). GC/MS: m/z 416, 418 [M+].

### *N*-[4-(5,6-dichloro-1*H*-benzo[*d*][1,2,3]triazol-1-yl)phenyl]-4-(trifluoromethyl)benzamide (7g)

C_20_H_11_Cl_2_F_3_N_4_O MW 451.23 Elem. Anal. Calc. C 53.24, H 2.46, N 12.42. Found C 53.64, H 2.68, N 12.75. Compound total yield 20%; m.p. > 290°C; TLC (petroleum ether/ethyl acetate 7/3): R_f_ 0.38. IR (nujol) ν: 1648, 3330 cm^−1^. UV (EtOH) λ_max_ 238, 281 nm. ^1^HNMR (DMSO): δ 10.82 (s, 1H, NH), 8.64 (s, 1H, H-4), 8.33 (s, 1H, H-7), 8.21 (d, *J* = 8.8 Hz, 2H, H-2′, 6′), 7.96 (d, *J* = 8.6 Hz, 2H, H-3′′, 5′′), 7.94 (d, *J* = 8.6 Hz, 2H, H-2′′, 6′′), 7.92 (d, *J* = 8.8 Hz, 2H, H-3′, 5′). ^13^C-NMR (DMSO): 164.7 (CO), 145.7 (C), 139.9 (2C), 136.6 (2C), 133.3 (C), 131.8 (2C), 131.4 (C), 129.8 (2CH), 128.5 (2CH), 123.4 (2CH), 121.3 (2CH), 121.0 (CH), 112.9 (CH). GC/MS: m/z 450, 452 [M+].

### *N*-[4-(5,6-dichloro-1*H*-benzo[*d*][1,2,3]triazol-1-yl)phenyl] 4-cyanobenzamide (7h)

C_20_H_11_Cl_2_N_5_O MW 408.24 Elem. Anal. Calc. C 58.84, H 2.72, N 17.15. Found C 58.44, H 2.38, N 17.47. Compound total yield 20%; m.p. 227–229°C; TLC (petroleum ether/ethyl acetate 7/3): R_f_ 0.58. IR (nujol) ν: 1674, 3350 cm^−1^. UV (EtOH) λ_max_ 208, 240 nm. ^1^HNMR (CDCl_3_/DMSO): δ 10.70 (s, 1H, NH), 8.36 (s, 1H, H-4), 8.19 (d, *J* = 8.8 Hz, 2H, H-2′, 6′), 8.10 (s, 1H, H-7) 8.14 (d, *J* = 8.6 Hz, 2H, H-3′′, 5′′), 7.91 (d, *J* = 8.6 Hz, 2H, H-2′′, 6′′), 7.79 (d, *J* = 8.8 Hz, 2H, H-3′, 5′). ^13^C-NMR (DMSO): 164.5 (CO), 145.7 (C), 139.2 (2C), 138.7 (2C), 132.5 (2CH), 133.3 (C), 131.8 (C), 128.6 (2CH), 123.4 (2CH), 121.3 (2CH), 119.6 (CH), 118.3 (CN), 114.3 (C), 110.9 (CH). GC/MS: m/z 407, 409 [M+].

### *N*-[4-(5,6-dichloro-2*H*-benzo[*d*][1,2,3]triazol-2-yl)phenyl]benzamide (8a)

C_19_H_12_Cl_2_N_4_O MW 383.23 Elem. Anal. Calc. C 59.55, H 3.16, N 14.62. Found C 59.65, H 3.40, N 14.32. Compound total yield 44%; m.p. > 290°C; TLC (petroleum ether/ethyl acetate 7/3): R_f_ 0.44. IR (nujol) ν: 1655, 3390 cm^−1^. UV (EtOH) λ_max_ 219, 345 nm. ^1^HNMR (CDCl_3_ /DMSO): δ 10.50 (s, 1H, NH), 8.28 (d, *J* = 9.0 Hz, 2H, H-2′, 6′), 8.24 (s, 2H, H-4, 7), 8.09 (d, *J* = 9.0 Hz, 2H, H-3′, 5′), 8.03-7.99 (m, 2H, arom.), 7.60-7.52 (m, 3H, arom). ^13^C-NMR (DMSO): 165.9 (CO), 140.3 (2C), 140.8 (C), 134.5 (2C), 131.8 (CH), 130.4 (2C), 128.4 (2CH), 127.7 (2CH), 120.9 (4CH), 119.5 (2CH). GC/MS: m/z 382, 384 [M+].

### *N*-[4-(5,6-dichloro-2*H*-benzo[*d*][1,2,3]triazol-2-yl)phenyl]-4-methylbenzamide (8b)

C_20_H_14_Cl_2_N_4_O MW 397.26 Elem. Anal. Calc. C 60.47, H 3.55, N 14.10. Found C 60.17, H 3.35, N 14.50. Compound total yield 35%; m.p. 210–213°C; TLC (petroleum ether/ethyl acetate 7/3): R_f_ 0.40. IR (nujol) ν: 1654, 3360 cm^−1^. UV (EtOH) λ_max_ 208, 350 nm. ^1^HNMR (CDCl_3_ /DMSO): δ 9.65 (s, 1H, NH), 8.30 (d, *J* = 8.6 Hz, 2H, H-2′, 6′), 8.08 (s, 2H, H-4, 7), 8.00 (d, *J* = 8.6 Hz, 2H, H-3′, 5′), 7.90 (d, *J* = 8.6 Hz, 2H, H-2′′, 6′′) 7.30 (d, *J* = 8.6 Hz, 2H, H-3′′, 5′′), 2.44 (s, 3H, CH_3_). ^13^C-NMR (DMSO): 165.9 (CO), 140.3 (2C), 140.8 (C), 134.5 (2C), 131.6 (C), 131.8 (2CH), 130.4 (2C), 128.4 (2CH), 127.7 (2CH), 120.9 (2CH), 119.5 (2CH), 21.0 (CH_3_). GC/MS: m/z 396, 398 [M+].

### *N*-[4-(5,6-dichloro-2*H*-benzo[*d*][1,2,3]triazol-2-yl)phenyl]-4-methoxybenzamide (8c)

C_20_H_14_Cl_2_N_4_O_2_ MW 413.26 Elem. Anal. Calc. C 58.13, H 3.41, N 13.56. Found C 58.48, H 3.61, N 13.18. Compound total yield 25%; m.p. 274–276°C; TLC (petroleum ether/ethyl acetate 7/3): R_f_ 0.56. IR (nujol) ν: 1,660, 3,370 cm^−1^. UV (EtOH) λ_max_ 212, 349 nm. ^1^HNMR (CDCl_3_ /DMSO): δ 10.15 (s, 1H, NH), 8.30 (d, *J* = 8.6 Hz, 2H, H-2′, 6′), 8.28 (s, 2H, H-4, 7), 8.10(d, *J* = 8.6 Hz, 2H, H-2′′, 6′′), 7.87 (d, *J* = 8.6 Hz, 2H, H-3′, 5′), 6.98 (d, *J* = 8.6 Hz, 2H, H-3′′, 5′′), 3.90 (s, 3H, OCH_3_). ^13^C-NMR (DMSO): 165.2 (CO), 162.1 (C-OCH_3_), 143.3 (2C), 141.1 (C), 134.3 (C), 130.4 (2C), 129.7 (2CH), 126.5 (C), 120.9 (4CH), 119.5 (2CH), 113.7 (2CH), 55.5 (OCH_3_). GC/MS: m/z 412, 414 [M+].

### *N*-[4-(5,6-dichloro-2*H*-benzo[*d*][1,2,3]triazol-2-yl)phenyl]-3,4,5-trimethoxybenzamide (8d)

C_22_H_18_Cl_2_N_4_O_4_ MW 473.31 Elem. Anal. Calc. C 55.83, H 3.83, N 11.84. Found C 56.00, H 4.13, N 11.44. Compound total yield 27%; m.p. 199–201°C; TLC (petroleum ether/ethyl acetate 7/3): R_f_ 0.75. IR (nujol) ν: 1,680, 3,360 cm^−1^. UV (EtOH) λ_max_ 215, 353 nm. ^1^HNMR (CDCl_3_): δ 10.00 (s, 1H, NH), 8.35 (d, *J* = 9.0 Hz, 2H, H-2′, 6′), 8.08 (s, 2H, H-4, 7), 7.86 (d, *J* = 9.0 Hz, 2H, H-3′, 5′), 7.11 (s, 2H, arom), 3.96 (s, 6H, 2OCH_3_), 3.93 (s, 3H, OCH_3_).). ^13^C-NMR (DMSO): 165.2 (CO), 152.6 (2C-O), 141.9 (C-O), 140.1 (C), 131.8 (2C), 131.7 (C), 131.2 (C), 130.8 (2C), 128.9 (2CH), 123.4 (2CH), 121.2 (2CH), 120.9 (CH), 112.9 (CH), 60.1 (OCH_3_), 56.1 (2OCH_3_). GC/MS: m/z 472, 474 [M+].

### *N*-[4-(5,6-dichloro-2*H*-benzo[*d*][1,2,3]triazol-2-yl)phenyl]-4-nitrobenzamide (8e)

C_19_H_11_Cl_2_N_5_O_3_ MW 428.23 Elem. Anal. Calc. C 53.29, H 2.59, N 16.35. Found C 53.00, H 2.19, N 16.65. Compound total yield 60%; m.p. > 297°C; TLC (petroleum ether/ethyl acetate 7/3): R_f_ 0.44. IR (nujol) ν: 1652, 3420cm^−1^. UV (EtOH) λ_max_ 216, 250, 345 nm. ^1^HNMR (CDCl_3_ /DMSO): δ 10.80 (s, 1H, NH), 8.42 (d, *J* = 8.6 Hz, 2H, H-2′, 6′), 8.20 (s, 2H, H-4, 7), 8.16 (d, *J* = 8.6 Hz, 2H, H-3′, 5′), 7.96 (d, *J* = 8.6 Hz, 2H, H-3′′, 5′′), 7.72 (d, *J* = 8.6 Hz, 2H, H-2′′, 6′′). ^13^C-NMR (DMSO): 166.9 (CO), 149.3 (C-NO_2_), 145.6 (2C), 140.3 (2C), 131.8 (C), 131.7 (C), 128.9 (2CH), 127.9 (2CH), 127.7 (C), 123.4 (2CH), 121.2 (2CH), 120.9 (2CH). GC/MS: m/z 427, 429 [M+].

### *N*-[4-(5,6-dichloro-2*H*-benzo[*d*][1,2,3]triazol-2-yl)PHENYL] 4-chlorobenzamide(8f)

C_19_H_11_Cl_3_N_4_O MW 417.68 Elem. Anal. Calc. C 54.64, H 2.65, N 13.41. Found C 54.34, H 2.85, N 13.80. Compound total yield 30%; m.p. 268–270°C; TLC (petroleum ether/ethyl acetate 7/3): R_f_ 0.34. IR (nujol) ν: 1654, 3270 cm^−1^. UV (EtOH) λ_max_ 262, 319 nm. ^1^HNMR (CDCl_3_ /DMSO): δ 10.72 (s, 1H, NH), 8.40 (d, *J* = 9.0 Hz, 2H, H-2′, 6′), 8.26 (s, 2H, H-4, 7), 8.20 (d, *J* = 9.0 Hz, 2H, H-3′, 5′), 8.04 (d, *J* = 8.6 Hz, 2H, H-3′′, 5′′) 7.62 (d, *J* = 8.6 Hz, 2H, H-2′′, 6′′). ^13^C-NMR (DMSO): 166.3 (C), 143.3 (2C), 140.6 (2C), 133.1 (2C), 130.5 (2C), 129.7 (2CH), 128.5 (2CH), 121.0 (4CH), 119.5 (2CH). GC/MS: m/z 416, 418 [M+].

### *N*-[4-(5,6-dichloro-2*H*-benzo[*d*][1,2,3]triazol-2-yl)phenyl]-4-(trifluoromethyl)benzamide (8g)

C_20_H_11_Cl_2_F_3_N_4_O MW 451.23 Elem. Anal. Calc. C 53.24, H 2.46, N 12.42. Found C 53.44, H 2.76, N 12.12. Compound total yield 65%; m.p. 210–212°C; TLC (petroleum ether/ethyl acetate 7/3): R_f_ 0.28. IR (nujol) ν: 1,660, 3,390 cm^−1^. UV (EtOH) λ_max_ 195, 254, 330 nm. ^1^HNMR (CDCl_3_ /DMSO): δ 9.80 (s, 1H, NH), 8.40 (d, *J* = 9.0 Hz, 2H, H-2′, 6′), 8.34 (s, 2H, H-4, 7), 7.95 (d, *J* = 8.6 Hz, 2H, H-3′′, 5′′) 7.61 (d, *J* = 9.0 Hz, 2H, H-3′, 5′), 6.70 (d, *J* = 8.6 Hz, 2H, H-2′′, 6′′). ^13^C-NMR (DMSO): 164.6 (CO), 144.4 (2C), 139.9 (2C), 138.4 (C), 135.3 (C), 133.5 (2C), 131.5 (CF_3_), 129.7 (2CH), 120.9 (4CH), 119.5 (2CH), 113.7 (2CH). GC/MS: m/z 450, 452 [M+].

### *N*-[4-(5,6-dichloro-2*H*-benzo[*d*][1,2,3]triazol-2-yl)phenyl] 4-cyanobenzamide (8h)

C_20_H_11_Cl_2_N_5_O MW 408.24 Elem. Anal. Calc. C 58.84, H 2.72, N 17.15. Found C 59.00, H 3.02, N 17.00. Compound total yield 90%; m.p. 263–265°C; TLC (petroleum ether/ethyl acetate 7/3): R_f_ 0.55. IR (nujol) ν: 1670, 3,400 cm^−1^. UV (EtOH) λ_max_ 250, 328 nm. ^1^HNMR (CDCl_3_ /DMSO): δ 10.93 (s, 1H, NH), 8.51 (s, 2H, H-4, 7), 8.30 (d, *J* = 8.6 Hz, 2H, H-2′, 6′), 8.17 (d, *J* = 8.6 Hz, 2H, H-3′, 5′), 7.98 (d, *J* = 8.6 Hz, 2H, H-3′′, 5′′), 7.83 (d, *J* = 8.6 Hz, 2H, H-2′′, 6′′). ^13^C-NMR (DMSO): 164.6 (CO), 144.4 (2C), 139.3 (C), 138.6 (C), 137.9 (2C), 135.5 (C), 132.5 (2CH), 128.6 (2CH), 121.1 (2CH), 120.4 (2CH).118.2 (C), 116.3 (2CH).113.9 (CN). GC/MS: m/z 407, 409 [M+].

### 1-[4-(5,6-dichloro-2*H*-benzo[*d*][1,2,3]triazol-2-yl)phenyl]-3-ethylurea (10a)

C_15_H_13_Cl_2_N_5_O MW 350.20 Elem. Anal. Calc. C 51.44, H 3.74, N 20.00. Found C 51.86, H 3.75, N 19.61. Compound total yield 52%; m.p. > 290°C; TLC (ether petroleum /ethyl acetate 7/3): R_f_ 0.17. IR (nujol) ν: 1630, 1615, 3300 cm^−1^. ^1^HNMR (DMSO): 8.91 (s, 1H, NH-ph), 8.45 (m, 2H, H-4, 7), 8.15 (d, J = 8.8 Hz, 2H, H-2′, 6′), 7.68 (d, *J* = 8.8 Hz, 2H, H-3′, 5′), 6.30 (t, *J* = 5.2 Hz, 1H, NH-CH_2_), 3.15 (q, *J* = 7.2 Hz, 2H, **CH**_**2**_CH_3_), 1.08 (t, *J* = 7.2 Hz, 3H, CH_2_**CH**_**3**_). ^13^C-NMR (DMSO): 154.9 (CO), 143.2 (2C), 142.5 (2C), 132.4 (C), 130.1 (C), 121.1 (2CH), 119.3 (2CH), 117.9 (2CH), 33.8 (CH_2_), 15.7 (CH_3_). LC/MS: m/z 372[M + Na], 352 [M + 1], 350 [M + 1].

### 1-[4-(5,6-dichloro-2*H*-benzo[*d*][1,2,3]triazol-2-yl)phenyl]-3-propylurea (10b)

C_16_H_15_Cl_2_N_5_O MW 364.23 Elem. Anal. Calc. C 52.76, H 4.15, N 19.23. Found C 52.46, H 4.50, N 19.40. Compound total yield 41%; m.p. > 300°C; TLC (ether petroleum /ethyl acetate 7/3): R_f_ 0.12. IR (nujol) ν: 1639, 1601, 3310 cm^−1^. ^1^HNMR (DMSO): 8.85 (s, 1H, NH-ph), 8.59 (s, H, H-4), 8.21 (s, H, H-7), 7.70 (s, 4H, H-2′, 3′, 5′, 6′), 5.80 (t, *J* = 5.2 Hz, 1H, NH-CH_2_), 2.90 (q, *J* = 7.2 Hz, 2H, **CH**_**2**_CH_3_), 1.49-1.22 (m, 2H, HN**CH**_**2**_), 0.89 (t, *J* = 7.2 Hz, 3H, CH_2_**CH**_**3**_). ^13^C-NMR (DMSO): 154.9 (CO), 143.2 (2C), 142.5 (2C), 132.4 (C), 130.1 (C), 121.1 (2CH), 119.3 (2CH), 117.9 (2CH), 40.9 (CH_2_NH), 22.9 (CH_2_), 11.3 (CH_3_). GC/MS: m/z 363, 365 [M+].

### 1-[4-(5,6-dichloro-2*H*-benzo[*d*][1,2,3]triazol-2-yl)phenyl]-3-butylurea (10c)

C_17_H_17_Cl_2_N_5_O MW 378.08 Elem. Anal. Calc. C 53.98, H 4.53, N 18.51. Found C 54.06, H 4.85, N 18.40. Compound total yield 61%; m.p. > 300°C; TLC (ether /petroleum ether 7/3): R_f_ 0.29. IR (nujol) ν: 1634, 1601, 3330 cm^−1^. ^1^HNMR (DMSO): 8.82 (s, 1H, NH-ph), 8.59 (s, H, H-4), 8.21 (s, H, H-7), 7.70 (s, 4H, H-2′, 3′, 5′, 6′), 6.26 (t, *J* = 5.2 Hz, 1H, NH-CH_2_), 3.13 (q, *J* = 7.4 Hz, 2H **CH**_**2**_CH_3_), 1.40–1.22 (m, 2H, 2**CH**_**2**_), 0.91 (t, *J* = 7.4 Hz, 3H, CH_2_**CH**_**3**_). ^13^C-NMR (DMSO): 154.9 (CO), 143.2 (2C), 142.5 (2C), 132.4 (C), 130.1 (C), 121.1 (2CH), 119.3 (2CH), 117.9 (2CH), 32.2 (CH_2_NH), 31.8 (CH_2_), 19.5 (CH_2_), 13.7 (CH_3_). LC/MS: m/z 400 [M + Na], 380 [M + 1], 378 [M + 1].

### 1-[4-(5,6-dichloro-2*H*-benzo[*d*][1,2,3]triazol-2-yl)phenyl]-3-cyclohexylurea (10d)

C_19_H_19_Cl_2_N_5_O MW 404.29 Elem. Anal. Calc. C 56.45, H 4.74, N 17.32. Found C 56.86, H 4.95, N 17.00. Compound total yield 25%; m.p. > 300°C; TLC (ether petroleum /ethyl acetate 8/2): R_f_ 0.72. IR (nujol) ν: 1,635, 1,584, 3,330 cm^−1^. ^1^HNMR (DMSO): 8.74 (s, 1H, NH-ph), 8.47 (s, 2H, H-4, 7), 8.16 (d, *J* = 8.0 Hz, 2H, H-2′, 6′), 7.65 (d, *J* = 8.0 Hz, 2H, H-3′, 5′), 6.24 (d, *J* = 5.2 Hz,1H, NH-CH_2_). 3.51 (m, 1H, CH), 1.84–1.85 (m, 2H, CH_2_), 1.69-1.66 (m, 2H, CH_2_), 1.57-1.54 (m, H, CH), 1.34-1.21 (m, 5H, 2CH_2_ and CH). ^13^C-NMR (DMSO): 154.0 (CO), 143.2 (C), 142.4 (C), 132.4 (C), 130.1 (C), 121.2 (2CH), 119.4 (2CH), 117.9 (2CH), 47.7 (CH), 32.8 (2CH_2_), 25.2 (CH), 24.3 (2CH). LC/MS: m/z 426 [M + Na], 406 [M + 1], 404 [M + 1].

### 1-[4-(5,6-dichloro-2*H*-benzo[*d*][1,2,3]triazol-2-yl)phenyl]-3-phenylurea (10e)

C_19_H_13_Cl_2_N_5_O MW 398.25 Elem. Anal. Calc. C 57.30, H 3.29, N 17.59. Found C 57.62, H 3.36, N 18.00. Compound total yield 69%; m.p. > 290°C; TLC (ether petroleum /ethyl acetate 6/4): R_f_ 0.66. IR (nujol) ν: 1,648, 1,593, 3,260 cm^−1^. ^1^HNMR (DMSO): 8.67 (s, 1H, NH), 8.39 (s, 2H, H-4, 7), 7.96 (d, *J* = 9.2 Hz, 2H, H-2′, 6′), 7.50 (d, *J* = 8.8 Hz, 2H, H-2′′, 6′′), 7.32-7.25 (m, 2H, H-3′′, 5′′), 7.01-6.97 (m, H, H-4′′), 6.74 (d, *J* = 8.8 Hz, 2H, H-3′, 5′). 5.85 (s, 1H, NH). ^13^C-NMR (DMSO): 152.5 (CO), 155.8 (2C), 143.0 (C), 139.7 (2C), 129.4 (C), 128.7 (4CH), 128.3 (C), 121.7 (CH), 118.9 (CH), 118.1 (4CH), 113.7 (CH). LC/MS: m/z 420 [M + Na], 400 [M + 1], 398 [M + 1].

### 1-[4-(5,6-dichloro-2*H*-benzo[*d*][1,2,3]triazol-2-yl)phenyl]-3-(4-methylphenyl)urea (10f)

C_20_H_15_Cl_2_N_5_O MW 412.27; Elem. Anal.: Calc. C 58.27, H 3.67, N 16.99. Found C 57.94, H 3.58, N 17.36. Compound total yield 57%; m.p. > 29 0°C; TLC (ether /petroleum ether 7/3): R_f_ 0.40. IR (nujol) ν: 1,714, 1,652, 3,350 cm^−1^. UV (EtOH) λ_max_ 208, 356 nm. ^1^HNMR (DMSO): 9.91 (s, 1H, NH), 9.42 (s, 1H, NH), 8.46 (s, 2H, H-4, 7), 8.21 (d, *J* = 8.4 Hz, 2H, H-2′, 6′), 7.74 (d, *J* = 8.4 Hz, 2H, H-3′, 5′), 7.37 (d, *J* = 7.6 Hz, 2H, H-2′′, 6′′), 7.10 (d, *J* = 7.6 Hz, 2H, H-3′′, 5′′), 2.25 (s, 6H, 2CH_3_). ^13^C-NMR (DMSO): 152.5 (CO), 143.2 (2C), 141.9 (C), 137.0 (C), 132,8 (C), 130.7 (C), 130.2 (2C), 129.2 (2CH), 121.2 (2CH), 119.4 (2CH), 118.2 (2CH), 118.1 (2CH), 20.3 (CH_3_). LC/MS: m/z 434 [M + Na], 414 [M + 1], 412 [M + 1].

### 1-[4-(5,6-dichloro-2*H*-benzo[*d*][1,2,3]triazol-2-yl)phenyl]-3-(4-methoxyphenyl)urea (10g)

C_20_H_15_Cl_2_N_5_O_2_ MW 428.27; Elem. Anal.: Calc. C 56.09, H 3.53, N 16.35. Found C 56.00, H 3.58, N 16.36. Compound total yield 53%; m.p. > 290°C; TLC (ether /petroleum ether 7/3): R_f_ 0.34. IR (nujol) ν: 1,730, 1,639, 3,310 cm^−1^. UV (EtOH) λ_max_ 207, 258, 356 nm. ^1^HNMR (DMSO): 9.96 (s, 1H, NH), 9.52 (s, 1H, NH), 8.98 (s, 2H, H-4, 7), 8.41 (d, *J* = 8.4 Hz, 2H, H-2′, 6′), 7.94 (d, *J* = 8.4 Hz, 2H, H-3′, 5′), 7.34 (d, *J* = 7.6 Hz, 2H, H-2′′, 6′′), 6.85 (d, *J* = 7.6 Hz, 2H, H-3′′, 5′′), 3.71 (s, 3H, OCH_3_). ^13^C-NMR (DMSO): 162.3 (CO), 152.0 (2C), 147.9 (2C), 136.4 (C), 134,4 (C), 130.7 (C), 130.2 (C), 129.2 (2CH), 121.6 (4CH), 119.2 (4CH), 55.1 (CH_3_). LC/MS: m/z 450 [M + Na], 430 [M + 1], 428 [M + 1].

### 1-[4-(5,6-dichloro-2*H*- benzo[*d*][1,2,3]triazol-2-yl)phenyl]-3-(4-chlorophenyl)urea (10h)

C_19_H_12_Cl_3_N_5_O MW 432.69; Elem. Anal.: Calc. C 52.74, H 2.80, N 16.19. Found C 53.00, H 2.58, N 16.36. Compound total yield 51%; m.p. > 290°C; TLC (ether/petroleum ether 7/3): R_f_ 0.29. IR (nujol) ν: 1,713, 1,651, 3,350 cm^−1^. UV (EtOH) λ_max_ 212, 255, 348 nm. ^1^HNMR (DMSO): 10.02 (s, 1H, NH), 9.81 (s, 1H, NH), 8.46 (s, 2H, H-4, 7), 8.21 (d, *J* = 8.0 Hz, 2H, H-2′, 6′), 7.74 (d, *J* = 8.0 Hz, 2H, H-3′, 5′), 7.52 (d, *J* = 8.0 Hz, 2H, H-2′′, 6′′), 7.34 (d, *J* = 8.0 Hz, 2H H-3′′, 5′′), 3.71 (s, 3H, OCH_3_). ^13^C-NMR (DMSO): 152.4 (CO), 143.2 (2C), 141.6 (C), 138.6 (C), 133,0 (C), 130.2 (2C), 128.6 (2CH), 125.4 (C), 121.2 (2CH), 119.5 (2CH), 119.4 (2CH), 118.3 (2CH). LC/MS: m/z 454 [M + Na], 434 [M + 1], 432 [M + 1].

### 1-[4-(5,6-dichloro-2*H*-benzo[*d*][1,2,3]triazol-2-yl)phenyl]-3-(4-fluorophenyl)urea (10i)

C_19_H_12_Cl_2_FN_5_O MW 416.24; Elem. Anal.: Calc. C 54.83, H 2.21, N 16.83. Found C 55.00, H 2.00, N 17.00. Compound total yield 62%; m.p. > 290°C; TLC (ether/petroleum ether 7/3): R_f_ 0.37. IR (nujol) ν: 1,727, 1,642, 3,350 cm^−1^. UV (EtOH) λ_max_ 209, 251, 347 nm. ^1^HNMR (DMSO): 9.62 (s, 1H, NH), 9.51 (s, 1H, NH), 8.45 (s, 2H, H-4, 7), 8.16 (d, *J* = 8.4 Hz, 2H, H-2′, 6′), 7.71 (d, *J* = 8.4 Hz, 2H, H-3′, 5′), 7.33-7.16 (m, 4H, H-2′′, 3′′, 5′′, 6′′). ^13^C-NMR (DMSO): 155.1 (CO), 143.2 (2C), 142.5 (2C), 140.1 (C), 132,4 (C), 130.1 (2C), 128.3 (2CH), 127.2 (2CH), 121.1 (2CH), 119.3 (2CH), 117.9 (2CH). LC/MS: m/z 434 [M + Na], 418 [M + 1], 416 [M + 1].

### 1-[4-(5,6-dichloro-2*H*-benzo[*d*][1,2,3]triazol-2-yl)phenyl]-3-benzylurea (10j)

C_20_H_15_Cl_2_N_5_O MW 412.27; Elem. Anal.: Calc. C 54.27, H 3.67, N 16.99. Found C 55.00, H 2.00, N 17.00. Compound total yield 49%; m.p. > 290°C; TLC (ether/petroleum ether 7/3): R_f_ 0.53. IR (nujol) ν: 1,627, 1,561, 3,400 cm^−1^. UV (EtOH) λ_max_ 210, 348 nm. ^1^HNMR (DMSO): 9.57 (s, 1H, NH), 8.46 (s, 2H, H-4, 7), 8.16 (d, *J* = 8.8 Hz, 2H, H-2′, 6′), 7.71 (d, *J* = 8.8 Hz, 2H, H-3′, 5′), 7.38-7.20 (m, 4H, H-2′′, 3′′, 4′′, 5′′, 6′′), 7.12 (t, *J* = 5.2 Hz, 1H, NHCH_2_), 4.33 (d, *J* = 5.2 Hz, 2H, CH_2_NH). ^13^C-NMR (DMSO): 152.6 (CO), 143.2 (2C), 141.8 (C), 135.9 (C), 132,9 (C), 130.2 (2C), 121.2 (2CH), 119.6 (CH), 119.5 (2CH), 118.3 (2CH), 115.4 (2CH), 115.2 (2CH). LC/MS: m/z 434 [M + Na], 414 [M + 1], 412 [M + 1].

### 1-[4-(5,6- dichloro-2*H*-benzo[*d*][1,2,3]triazol-2-yl)phenyl]-3-naphtylurea (10k)

C_23_H_15_Cl_2_N_5_O MW 448.30; Elem Anal.: Calc. C 61.62, H 3.37, N 15.62. Found C 62.00, H 2.98, N 16.90. Compound total yield 47%; m.p. > 290°C; TLC (petroleum ether/ethyl acetate 7/3): R_f_ 0.71. IR (nujol) ν: 1,635, 1,561, 3,260 cm^−1^. ^1^HNMR (DMSO): 9.20 s, (2H, NH), 8.25 (d, *J* = 8.0 Hz, 2H, H-2′, 6′), 8.10 (d, *J* = 8.0 Hz, 2H, H-3′, 5′), 8.03-7.90 (m, 2H, Ph), 7.78 (s, 2H, H-4, 7), 7.75-7.40 (m, 5H, Ph). ^13^C-NMR (DMSO): 154.7 (CO), 153.3 (2C), 152.8 (C), 143.2 (C), 141,6 (C), 134.3 (2C), 133.7 (2C), 128.4 (2CH), 126.1 (CH), 125.9 (2CH), 125.6 (2CH), 122.9 (2CH) 121.4 (2CH). 117.5 (2CH). LC/MS: m/z 470 [M + Na], 450 [M + 1], 448 [M + 1].

## Biological Assay Descriptions

### Cells and Viruses

Cell lines were purchased from American Type Culture Collection (ATCC). The absence of mycoplasma contamination was checked periodically by the Hoechst staining method. Cell lines supporting the multiplication of RNA and DNA viruses were the following: CD4+ human T-cells containing an integrated HTLV-1 genome (MT-4); Madin Darby Bovine Kidney (MDBK) [ATCC CCL 22 (NBL-1) *Bos Taurus*]; Baby Hamster Kidney (BHK-21) [ATCC CCL 10 (C-13) *Mesocricetus Auratus*]; monkey kidney (Vero-76) [ATCC CRL 1587 *Cercopithecus Aethiops*] and human epithelial cell lines (human laryngeal carcinoma) HEp-2 (HEp-2) [ATCC CCL-23)].

Viruses were purchased from American Type Culture Collection (ATCC), with the exception of Human Immunodeficiency Virus type-1 (HIV-1) and Yellow Fever Virus (YFV). Viruses representative of positive-sense, single-stranded RNAs (ssRNA+) were: (i) *Retroviridae*: the III_B_ laboratory strain of HIV-1, obtained from the supernatant of the persistently infected H9/III_B_ cells (NIH 1983); (ii) *Flaviviridae*: Yellow Fever Virus (YFV) [strain 17-D vaccine (Stamaril Pasteur J07B01)] and Bovine Viral Diarrhea Virus (BVDV) [strain NADL (ATCC VR-534)]; (iii) *Picornaviridae*: Human Enterovirus B [coxsackie type B5 (CV-B5), strain Faulkner (ATCC VR-185)] and Human Enterovirus C [poliovirus type-1 (Sb-1), Sabin strain Chat (ATCC VR-1562)]. Viruses representative of negative-sense, single-stranded RNAs (ssRNA-) were: (iv) *Pneumoviridae*: Human Respiratory Syncytial Virus (RSV) strain A2 (ATCC VR-1540); RSV strain B1 *cp-52* clone 2B5 (ATCC-VR-2542); (v) *Rhabdoviridae*: Vesicular Stomatitis Virus (VSV) [lab strain Indiana (ATCC VR 1540)]. The virus representative of double-stranded RNAs (dsRNA) was: (vi) *Reoviridae* Reovirus type-1 (Reo-1) [simian virus 12, strain 3651 (ATCC VR-214)]. DNA virus representatives were: (vii) *Poxviridae*: Vaccinia Virus (VV) [vaccine strain Elstree-Lister (ATCC VR-1549)]; (viii) *Herpesviridae*: Human Herpes 1 (HSV-1) [strain KOS (ATCC VR-1493)].

Viruses were maintained in our laboratory and propagated in appropriate cell lines. All viruses were stored in small aliquots at −80°C until use.

### Cytotoxicity Assays

Exponentially growing MT-4 cells were seeded at an initial density of 4x10^5^ cells/mL in 96-well plates containing RPMI-1640 medium, supplemented with 10% fetal bovine serum (FBS), 100 units/mL penicillin G and 100 μg/mL streptomycin. MDBK, Hep-2 and BHK cells were seeded at an initial density of 6 × 10^5^, 5 × 10^5^, and 1 × 10^6^ cells/mL, respectively, in 96-well plates containing Minimum Essential Medium with Earle's salts (MEM-E), L-glutamine, 1 mM sodium pyruvate and 25 mg/L kanamycin, supplemented with 10% horse serum (MDBK) or 10% fetal bovine serum (FBS) (BHK; Hep-2). Vero-76 cells were seeded at an initial density of 5 × 10^5^ cells/mL in 96-well plates containing in Dulbecco's Modified Eagle Medium (D-MEM) with L-glutamine and 25 mg/L kanamycin, supplemented with 10% FBS. Cell cultures were then incubated at 37°C in a humidified, 5% CO_2_ atmosphere, in the absence or presence of serial dilutions of test compounds. The test medium used for cytotoxic and antiviral assay contained 1% of the appropriate serum. Cell viability was determined after 72, 96, or 120 h at 37°C by the MTT method for MT-4, MDBK, Hep-2, BHK and Vero-76 cells (Budge et al., 2004).

### Antiviral Assays

Compounds' activity against HIV-1 was based on inhibition of virus-induced cytopathogenicity in exponentially growing MT-4 cells acutely infected with a multiplicity of infection (m.o.i.) of 0.01. Antiviral activity against YFV and Reo-1 was based on inhibition of virus-induced cytopathogenicity in BHK-21 cells acutely infected with a m.o.i. of 0.01. Compounds' activity against BVDV was based on inhibition of virus-induced cytopathogenicity in MDBK cells acutely infected with a m.o.i. of 0.01. After a 3 or 4-day incubation at 37°C, cell viability was determined by the MTT method as described by Pauwels et al. (1988). Compounds' activity against CV-B5, Sb-1, VSV, VV, RSV A2 and HSV-1 was determined by plaque reduction assays in infected cell monolayers, as described by Sanna et al. (2015).

### Virucidal Activity Assay

**10d** (20 μM) was incubated with 1x10^5^ PFU/mL of RSV at either 4 and 37°C for 1 h. The mixture without test sample was used as the control. At the end of incubation period, samples were serially diluted in media and titers were determined on Vero-76 cells at high dilutions, at which the compound was not active. Virus titers were determined by plaque assay in Vero-76 cells.

### Assessment of Antiviral Activity by Antigen Reduction Assay

**10d**, 6-azauridine and dextran sulfate were diluted by serial dilutions in MEM-E to give a range of compounds concentrations from 100 to 0.8 μM. 50 μL of RSV (approximately 200 PFU/well) and 50 μL of compounds dilutions was added in triplicate to Vero-76 cells in 96 well plates that had been seeded the previous day at 2.5 × 10^4^ cells per well. At 3 days post infection, samples were collected and the extent of viral replication was determined by enzyme-linked immunosorbent assay (ELISA) according to the manufacturer description (Bioo Scientific RSV Kit).

### Cell Pretreatment Assay

Vero-76 cell monolayers were incubated in 24-well plates with 20 μM concentration of **10d** or 6-azauridine (10 μM) for 2 h at 4°C. After removal of the compound and two gentle washes, cells were infected with RSV. After virus adsorption to cells, the inoculum was removed and the cells were then overlaid with medium, incubated for 3 days at 37°C, and then virus titers were determined by plaque assay.

### Time of Addition Assay

The confluent monolayers of Vero-76 cells in 96-well tissue culture plates were infected for 2 h at room temperature with 100 μL of proper RSV dilutions to give a final m.o.i. of 1. After adsorption, the monolayers were washed two times with MEM-E medium with L-glutamine, supplemented with 1% inactivated FBS, 1 mM sodium pyruvate and 0.025 g/L kanamycin (Maintenance Medium) and incubated with the same medium at 5% CO_2_ and 37°C (time zero). Vero-76 cells were treated with compound **10d** (20 μM, approximately 10 times higher than the IC_50_) or reference for 2 h during infection period and at specific time point, 2, 4, 6, and 10 h post infection. After each incubation period, the monolayers were washed two times with maintenance medium and incubated with fresh medium until 10 h post-infection. Then, after 36 h the development of cytopathic effect (CPE) was evaluated microscopically and each sample was collected, centrifuged and frozen at −80°C. The viral titer was determined by plaque assay (or ELISA).

### Immunofluorescence Attachment Assay

Compounds' inhibitory effects on virus-cell binding event was measured by immunofluorescence performed according to the method described previously with some modifications (Maric et al., 2011). Briefly, Vero-76 cells in 24-well plates were pre-chilled for 1 h at 4°C. The medium was removed and then serial dilutions of RSV in ice-cold MEM-E were added and allowed to bind for approximately 30 min at 4°C. After this incubation period, the monolayers were washed three times in cold PBS in order to remove unbound viral particles and then were fixed in paraformaldehyde 4%. The presence of bound virus was detected by indirect immunofluorescence. After a brief incubation in blocking buffer [PBS with 5% normal goat serum (NGS)], cells were incubated with a 1:200 dilution of primary antibody RSV monoclonal antibody (Ab35958; Abcam, Cambridge, United Kingdom). Cells were then washed three times in PBS-Tween-100 and incubated with a 1:500 dilution of an Alexa Fluor 488-labeled anti-mouse secondary antibody (Molecular Probes, Eugene, Oreg.). After a series of washes in PBS-Tween-100, cells were mounted with Vectashield (Vector Laboratories, Burlingame, CA) containing 4′,6-diamidino-2-phenylindole (DAPI) and images of each well were captured with the ZOE Fluorescent Cell Imager (Biorad).

### Inhibition of Attachment Assessed by Immunofluorescence

RSV viral dilution (4 × 10^6^ PFU/mL) was premixed with 20 μM of **10d**, 6 μg/mL of dextran sulfate, and 5 μM of 6-azauridine in ice-cold MEM, added to Vero-76 cell monolayers at 4°C, bound, and detected as reported above.

### Syncytium Reduction Assay

The capability of **10d** to block cell-to-cell RSV spread was evaluated using the above described method. Monolayers cultures of Vero-76 cells in six-well dishes were infected with RSV (m.o.i. = 0.3) at room temperature. After 2 h of adsorption period, the monolayer was washed with PBS and overlaid with fresh medium. **10d** was added at concentration of 50, 10, and 2 μM at 8 h post infection. 72 h post infection cells were examined microscopically for syncytium formation.

### RSV Strain B1 cp-52 Antiviral Assay

The effect of **10d** on RSV cp-52 plaque number was assessed by a plaque reduction assay. Accordingly, a monolayer of Vero-76 cells was grown on 24-well plate. Approximate 200 plaque-forming units (PFU) of RSV cp-52 was added to the cells, immediately followed by the addition of various concentrations of the samples. The medium was also added to non-treatment wells as non-infection controls. The plates were incubated in 5% CO_2_ at 37°C for 2 h. After virus adsorption the inoculum was removed and infected cells were overlaid with 1.2% methylcellulose medium containing various concentrations of test samples and incubated for 3 days at 37°C. Syncytia developed after 3 days and were fixed with 4% paraformaldehyde solution, permeabilized and immunostained. The number of plaques in the control (no inhibitor) and experimental wells were counted.

### End-point Cell-to-Cell Fusion Assay

The 293T cell line was purchased from the American Type Culture Collection (Manassas, VA) and maintained in DMEM supplemented with 7.5 (v/v) FBS at 37°C and 5% CO_2_. Cell transfection was carried out using Lipofectamine 200 (Invitrogen). A dual split-protein cell content mixing assay was adopted to quantify the extent of cell-to-cell fusion mediated by the RSV F-glycoprotein. To the purpose, 293T cells were transfected with plasmid DNA encoding the eGFP-renilla luciferase dual-split fusion proteins DSP_1−7_ or DSP_8−11_, respectively (Kondo et al., 2011). Then, one cell population received the plasmid DNA encoding RSV L19F while another cell population was transfected with plasmid DNA coding for Mev F and H proteins for control (Brindley et al., 2012). According to the protocol of Yan et al. (2014) cell populations were mixed at an equal ration 4 h after transfection, and incubated with specified amount of 10d for 26 hrs. The activity of the reconstituted luciferase was measured after cell loading with 10 μM ViviRen (Promega) for 30 min.

### *In silico* Molecular Modeling

All simulations were performed with the AMBER 16 suite of programs (Case et al., 2017). The 3D model structure of the prefusion RSV F glycoprotein was taken from the Protein Data Bank (file 5EA5.pdb (Battles et al., 2016). Compound **10d** was then docked into the protein-binding site using Autodock 4.2 (Morris et al., 2016). The resulting complex was further energy minimized to convergence. The intermolecular complex was then solvated and energy minimized using a combination of molecular dynamics (MD) techniques (Pierotti et al., 2011; Gibbons et al., 2014; Genini et al., 2017). 20 ns molecular dynamics (MD) simulations at 37°C were then employed for system equilibration, and further, 50-ns MD were run for data production.

The binding free energy, ΔG_bind_, between **10d** and the RSV F-protein was then estimated by resorting to the MM/PBSA approach (Massova and Kollman, 2000) implemented in Amber 16. According to this validated methodology, the free energy was calculated for each molecular species (complex, protein, and ligand), and the corresponding ΔG_bind_ was computed as:

$$\begin{array}{ll} \Delta G_{\text{bind}}=G_{\text{complex}}-\left(G_{\text{protein}}+G_{\text{ligand}}\right)\;=\;\Delta E_{\text{MM}}+\Delta G_{\text{SOL}}\\ - & T\Delta S_{\text{bind}}\;=\;\Delta H_{\text{bind}}-T\Delta S_{\text{bind}} \end{array}$$

in which the enthalpic contribution (ΔH_bind_ = ΔE_MM_ + ΔG_SOL_) accounts for the molecular mechanics energy (contributed by van der Waals and electrostatic interactions) and the solvation free energy while TΔS is the conformational entropy upon protein/ligand binding. The corresponding IC_50_ value for each compound was obtained from the fundamental relationship: ΔG_bind_ = -RT log 1/IC_50_. The same computational protocol was applied for the simulation of TMC353121, an established potent RSV F-protein inhibitor, in complex with the RSV F-protein for comparison purposes.

## Result and Discussion

### Chemistry

The synthetic routes to obtain 5,6-dichloro-1-phenyl-benzotriazole amides (**5a-d** and **7a-h**), 5,6-dichloro-2-phenyl-benzotriazole amides (**6a-h** and **8a-h**), and 5,6-dichloro-2-phenyl-benzotriazole urea derivatives **(10a-k)** are described in Schemes 1–3, respectively. Aliphatic amides **5a-d** and **6a-f** were obtained in good yield by condensation of the known anilines **1a,b** with the appropriate anhydride (acetic, propionic, and butyric anhydride) (**2a-c**) at 100°C for 1 h. Derivatives **5c** and **6a-c** were obtained together to their respective diacylated compounds **5d, 6d-f**, generally in 3:1 ratio. Aromatic amides **7a-h** and **8a-h** were in turn obtained, generally in good yield, by reaction of the anilines **1a,b** with the appropriate benzoyl chloride derivative (**3a-h**) at 80°C for 0.5–72 h. To obtain the urea derivatives (**10a-k**) (35–70% yield,) the required isocyanate (**4a-k**) was condensed with aniline **1b** at 80–110°C for 24–92 h.

After workup, all crude products were purified by recrystallization with ethanol or by flash chromatography. The key intermediates 1-(4-aminophenyl)-5,6-dichlorobenzotriazole (**1a)** and 2-(4-aminophenyl)-5,6-dichlorobenzotriazole **(1b**) were prepared following the procedure described by Carta et al. (2007).

### *In vitro* Antiviral Activity of the New Dichloro-phenyl-benzotriazoles

All 39 newly synthesized 5,6-dichloro-1(2)-phenyl-benzotriazole derivatives were evaluated for their anti-RSV activity in cell-based assays. Several compounds exhibited significant inhibitory activity (i.e., <10 μM, Table 1), with EC_50_ values lower than or comparable to that of ribavirin (7 μM). Concomitantly, moderate to low cytotoxicity was detected for almost all compounds, with CC_50_ values mostly in the high micromolar range (> 100 μM) in Vero-76 cells.

**Table 1:** Anti-RSV activity of 5,6-dichloro-1-phenyl-benzotriazole amides (**5a–d** and **7a–h**), 5,6-dichloro-2-phenyl-benzotriazole amides (**6a–h** and **8a–h**), and 5,6-dichloro-2-phenyl-benzotriazole ureas (**10a–k**).

**Comp**.	**Vero-76 cells**	**RSV**	**Comp**.	**Vero-76 cells**	**RSV**
	**^**a**^ CC_**50**_**	**^**b**^EC_**50**_**		**^**a**^*CC*_50_**	**^**b**^*EC*_50_**
**1a**	>100	**20**	**8a**	>100	>100
**5a**	30	**3**	**8b**	>100	**27**
**5b**	30	**4**	**8c**	>100	>100
**5c**	10	**4**	**8d**	80	**3**
**5d**	20	>30	**8e**	>100	**33**
**7a**	>100	60	**8f**	80	**20**
**7b**	>100	>100	**8g**	>100	50
**7c**	>100	**35**	**8h**	>100	60
**7d**	90	**5**	**10a**	>100	>100
**7e**	>100	**13**	**10b**	30	**3**
**7f**	>100	**8**	**10c**	>100	>100
**7g**	9	**5**	**10d**	90	**2**
**7h**	>100	**11**	**10e**	>100	>100
**1b**	>100	**6**	**10f**	>100	>100
**6a**	>100	>100	**10g**	>100	>100
**6b**	>100	>100	**10h**	>100	>100
**6c**	>100	>100	**10i**	>100	>100
**6d**	>100	**40**	**10j**	>100	>100
**6e**	>100	**6**	**10k**	>100	>100
**6f**	>100	**6**	**ribavirin**	>100	**7**
			**6-azauridine**	≥14	**1.4**

*Ribavirin and 6-azauridine were used as reference inhibitors. Data represent mean values from three independent determinations; variation among duplicate samples was <15%*.

a *Compound concentration (μM) required to reduce the viability of mock-infected VERO-76 cells by 50%. as determined by the MTT method after 5 days*.

b *Compound concentration (μM) required to reduce the plaque number of RSV by 50% in VERO-76 monolayers. The most interesting results are in bold*.

Regarding the spectrum of antiviral activity, the most relevant result concerned the potent and selective activity of ten 1-phenyl-benzotriazole derivatives (**1a**, **5a**,**b,c** and **7c-h**), seven 2-phenyl-benzotriazole derivatives (**1b**, **6d**,**e**,**f**, **8b**,**d**,**e,f**), and two 2-phenyl-benzotriazole urea derivatives (**10b**,**d**), with an EC_50_ against RSV in a range of 2–40 μM.

From a structure-activity relationship perspective, considering the 1-phenyl-benzotriazole amides the replacement of a hydrogen atom on the amino group with any aliphatic moiety (i.e., methyl, ethyl or propyl) in the amidic component (**1a** and **5a-c**, respectively) did reflect in a good increase in potency, even if higher potencies were coupled with higher cytotoxicity (EC_50_ values 20 vs. 3/4 μM, CC_50_ values >100 vs. 10/30 μM, Table 1). On the contrary, the presence of two acyl substituents on the amino group (**5d**) was detrimental to antiviral activity. In essence, these results demonstrate the need for a hydrogen atom on the amide nitrogen for RSV replication inhibition. Conversely, in the case of aromatic amides the unsubstituted phenyl derivative (**7a**) showed only a moderate activity (EC_50_ = 60 μM) whereas the introduction of three methoxy groups on the phenyl moiety (**7d**) or an electron-withdrawing group in the 4' position (**7e-h**) potentiated anti-RSV activity (EC_50_ = 5–35 μM, Table 1). On the other hand, an electron-donor methyl group in the same position (**7b**) led to a pitfall of the corresponding EC_50_ value. Finally, for the series of 5,6-dichloro-1-phenyl-benzotriazole amides a small alkyl group on the nitrogen gave the best antiviral activity, while its substitution with a phenyl ring was only useful if an electron-withdrawing or three methoxy groups were presents.

Also, the 2-phenyl-benzotriazole amides showed a diffuse antiviral activity against RSV. Amine (**1b**), aliphatic amides (**6d,e,f**) and aromatic amides (**8b,d,e,f**) showed activity in the 3–40 μM range. Contrarily to the case of **1**-phenyl derivatives, for the 2-phenyl ones the best activity of the diacyl derivatives (**6d,e,f**) compared to that of the corresponding monoacyl compounds (**6a,b,c**) was probably due to a different arrangement in the space of the amide chains. Furthermore, also in this series the simple unsubstituted phenyl amide or 4' substitution with an electron-donor group (**8a** and **8b,c**, respectively) generally did not exhibit high activity (EC_50_ = 27 - >100 μM), while the derivative bearing three methoxy groups or a nitro group or a chlorine atom in 4' on the phenyl moiety (**8d** and **8e**,**f**, respectively) mostly showed good activity (EC_50_ = 3–33 μM, Table 1). Finally, about the 2-phenyl-benzotriazole urea derivatives (**10a-k**) only two aliphatic derivatives showed a remarkable activity against RSV (**10b,d**), with EC_50_ values of 3 and 2 μM, respectively.

With the aim of evaluating the selectivity of the title compounds against RSV, they were also tested for cytotoxicity and antiviral activity against a panel of alternative, representative positive- and negative-sense single stranded RNA, double-stranded RNA and DNA viruses (see Tables S1, S2 in Supplementary Material). Among all 39 compounds, **5a**,**c** and **8c** were moderately active only against Coxsackie Virus B5 (CV-B5, EC_50_ = 17, 9 and 14 μM, respectively), while **8e,h** showed some activity in cells infected with the Bovine Viral Diarrhea Virus (BVDV, EC_50_ = 4 and 11 μM, respectively). Concomitantly, the overall low cytotoxic profile of all inhibitors was confirmed in the cell lines sustaining the alternative virus replication (Tables S1, S2).

Based on the results of all experiments described above, **10d** resulted the most interesting compound, being endowed with potent and selective antiviral activity against RSV (EC_50_ = 2 μM, Table 1) and CC_50_ values lower than 70 μM in most continuous cell lines from different organs and species (Table 1, Table S2). Therefore, **10d** was selected for additional studies, as reported below.

## Biological and *in Silico* Evaluation

### Viral Antigen Reduction by Compound 10d

The antiviral activity of **10d** was further tested in an enzyme-linked immunosorbent assay (ELISA) aimed at demonstrating the reduction of viral antigen in presence of the active compound at non-cytotoxic concentrations during a single round of viral infection. Results showed a reduction of viral antigen of 80% at 20 μM and an EC_50_ of 5μM, in agreement with previous plaque reduction assay data.

### Virucidal Activity of 10d

**Figure 1:**
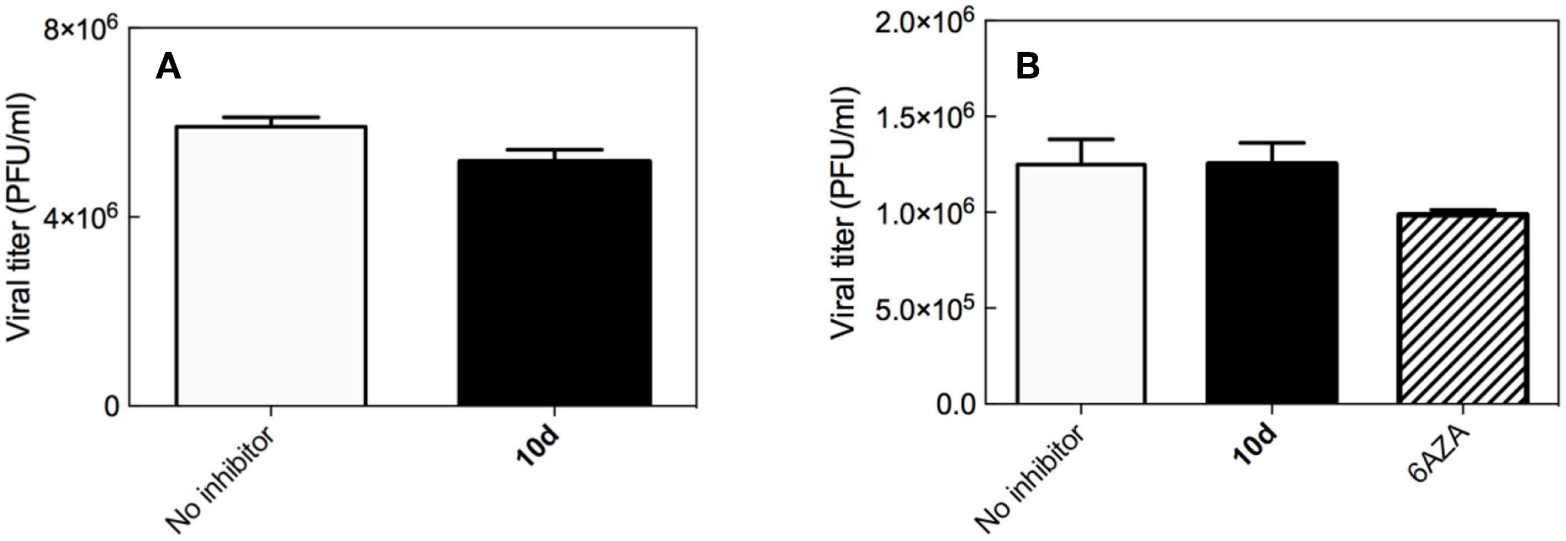
**(A)** Virucidal effect (expressed as plaque-forming units (PFU)/mL) of **10d** (20 μM) against RSV virions at either 4 or 37°C) for 1 h. **(B)** Viral titer measured 3 days post infection in Vero-76 cells pretreated with 10 d. The same experiment performed with 6-azauridine (6AZA 10 μM) is shown for comparison. Results are presented as mean ± standard deviation of 3 separate experiments.

To analyze the possibility that compound **10d** acts directly on the virus particle leading to infectivity inactivation, a virucidal assay against RSV virions was conducted. The virucidal effect of **10d** was negligible at the tested concentration of 20 μM at either 4°C or 37°C and no significant differences between the titer of RSV treated at the two different temperatures was observed, as shown in Figure 1A. The compound tested concentration (20 μM) was 10 times higher than its antiviral EC_50_, indicating that the inhibitory effect detected by the plaque reduction assay (2 μM, Table 1) could be due to interference with a step along the RSV replication cycle.

### Effect of 10d on RSV Penetration into Pre-treated Host Cells

To establish whether **10d** was able to protect cells from RSV infection, a pre-attachment assay was then performed by incubating Vero-76 cell monolayers with the same concentration of **10d** employed in the virucidal activity assay (20 μM). 6-Azauridine (10 μM), a broad spectrum DNA/RNA inhibitor that interferes in pyrimidine biosynthesis pathway, was used as negative reference compound. Eventual unbound drug was washed away, cells were infected with RSV, and percent of infection was determined after 3 days. The results in Figure 1B shows that, under these experimental conditions, **10d** failed to inhibit RSV infection at the time point analyzed. These findings demonstrate that pretreatment with **10d** does not protect cells from RSV infection.

### Effect of 10d on Viral Infection: Time of Addition and Immunofluorescence Assays

**Figure 2:**
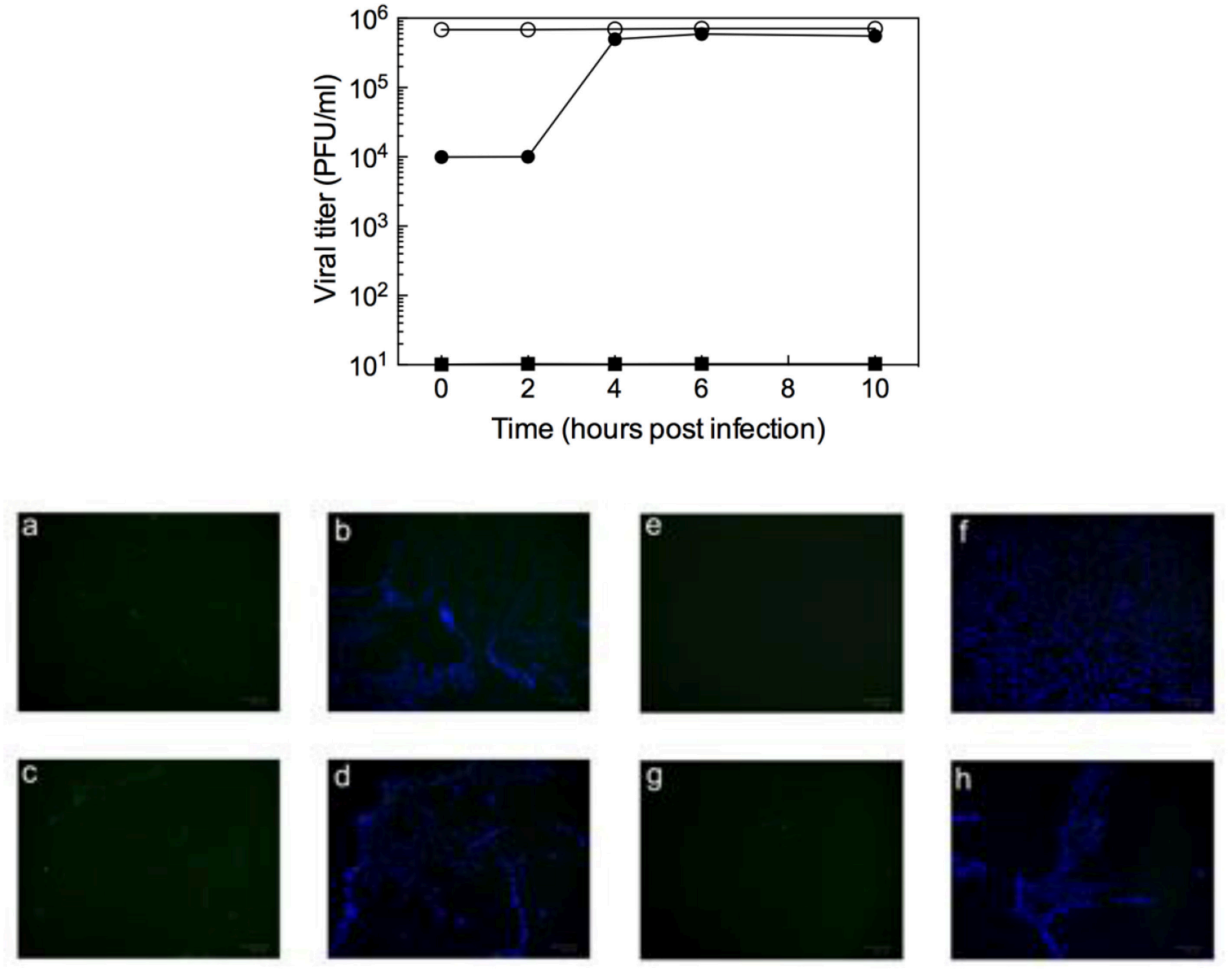
**(Top)** Inhibition of RSV (m.o.i = 1) by addition of **10d** (20 μM) at different times (filled circles). Data for untreated virus (open circles) and for addition of 6-azauridine (filled squares) are also shown for comparison. Data represent mean values from two independent determinations; variation among duplicate samples was <15%. **(Bottom)** Immunofluorescence attachment assay: (a,b) RSV A2 non-treated control; (c,d) 6-azauridine does inhibit viral attachment; (e,f) dextran sulfate inhibits viral attachment; (g,h) **10d** does not inhibit viral attachment. Bound virus is detected by indirect immunofluorescence with an anti-G monoclonal antibody, followed by an Alexa Fluor 488-labeled secondary antibody (a,e,c,g, green). Nuclei are visualized using 4′,6-diamidino-2-phenylindole (b,f,d,h, blue).

To determine which step of the RSV replication cycle was targeted by **10d**, time of addition (ToA) experiments were performed in RSV-infected Vero-76 cells exposed to the compound at different times of infection. As seen in the top panel of Figure 2, the most efficient inhibition was observed in early phases of infection [i.e., during the 2 h of infection (*t* = 0) and immediately after], while **10d** failed to exhibit significant antiviral activity when added at later times (4–10 h after infection). These results (supported by additional ToA tests performed with two further, active compounds, **8d** and **10b**, EC_50_ = 3 μM, Table 1, see Figure S1 in Supplementary Material) confirm that **10d** is active in the earliest stages of viral replication, that is the virus attachment to target cells or the virus-cell fusion event.

In the attempt to discriminate between the two possible mechanisms of action of **10d** (i.e., virus attachment or cell-fusion inhibition), the kinetics of virus adsorption in the presence **10d** was investigated in an immunofluorescence assay. Low-temperature treatment allows binding of RSV to the cell surface receptors but prevents the internalization of virus particles into the cells. Accordingly, the ability of **10d** to interfere with virus binding was examined by incubating RSV and Vero-76 cells at 4°C in the presence of the compound. As reported in the bottom panel of Figure 2, **10d** and 6-azauridine (negative control) were not able to block viral binding under these conditions. On the contrary, dextran sulfate, an RSV entry inhibitor used as a positive control, efficiently blocked viral attachment.

In aggregate, these results led us to speculate that either the virus-cell fusion or the uncoating processes might be affected by the antiviral action of **10d**.

### Effect of 10d on the Formation of Multinucleate Syncytia

**Figure 3:**
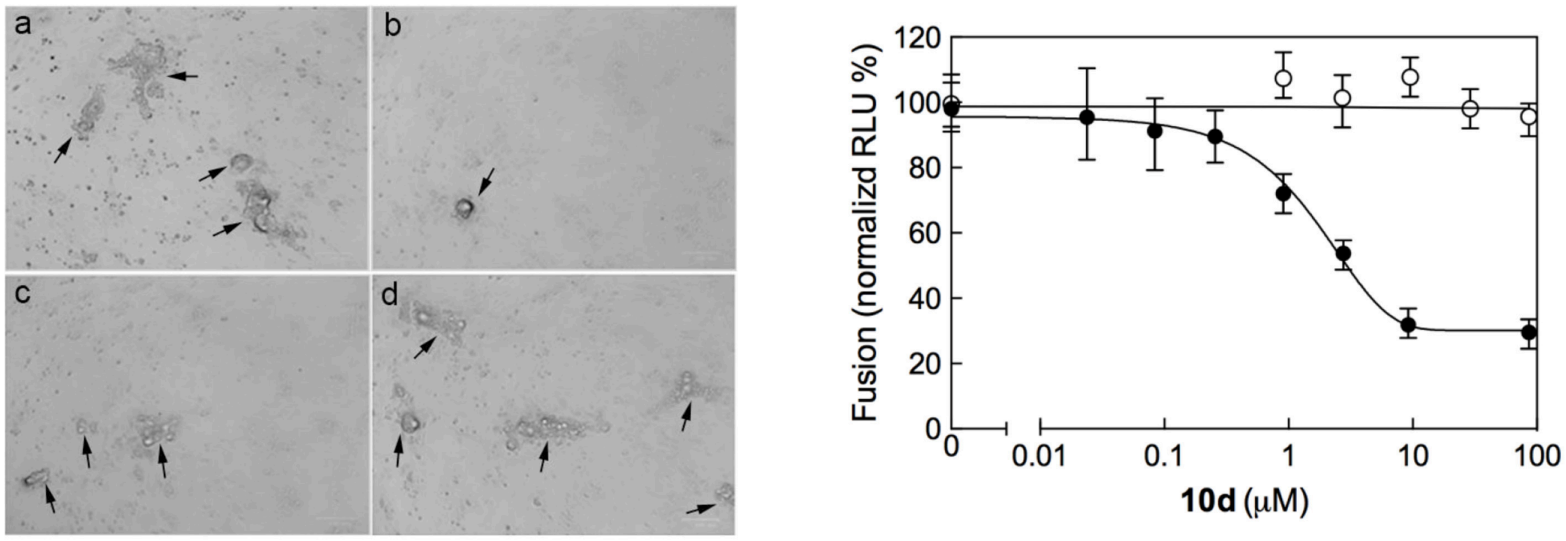
**(Left)** Inhibition of RSV A2-induced syncytium formation in Vero-76 cells by **10d** at a concentration of 0 μM (a), 50 μM (b), 10 μM (c), and 2 μM (d). Inoculum was removed 2 h post infection (p.i.), cells were left untreated or incubated 8 h p.i. with **10d**, and syncytia formation was assessed 72 h p.i. as described in Materials and Methods. In the presence of **10d** both size and number of syncytia did not increase. Images were taken 72 h p.i. using the ZOE Fluorescent cell imager (Bio-Rad) (bar size = 100 μm). Syncytia are indicated by black arrows. **(Right)** Quantitative dose-response cell-to-cell fusion assay using the DSP-chimeric reporter proteins and the ViviRen renilla luciferase substrate in the presence of compound **10d** (filled symbols). The MeV (Measles Virus) F and H glycoprotein expression constructs (open symbols) were included for selectivity control. Reported values are normalized for DMSO-treated samples and are expressed as the mean of three experiments ± standard deviation. The EC_50_ value was obtained by 4-parameter variable slope regression fitting.

Syncytium formation is a mechanism of cell-to-cell infection that determines virus spread. Common features of severe lower respiratory infections with RSV are cell fusion, multinucleate syncytia formation, and cell sloughing. In order to ascertain whether **10d** was able to prevent virus spread after infection, a syncytium reduction assay was performed by treating Vero-76 monolayers with different concentrations of **10d** at 8 h post infection (p.i). Syncytia formation was assessed 72 h p.i. The left panel in Figure 3 shows that big syncytia were evident in Vero-76 untreated monolayers after 72 h p.i. (a), while the number and the size of syncytia decreased in a dose-dependent manner in **10d** treated cells (b, c, and d).

The initial phases of RSV infections are controlled by the two major glycoproteins on the surface of the RSV virion that is, the attachment glycoprotein (G) and the fusion glycoprotein (F). G targets the ciliated cells of the airways, and F causes the virion membrane to fuse with the target cell membrane. The small hydrophobic (SH) protein is also encoded by the human respiratory syncytial virus. Its absence leads to viral attenuation in the context of whole organisms, and it prevents apoptosis in infected cells (Heminway et al., 1994; Kahn et al., 1999; Techaarpornkul et al., 2001). Among this protein pool, however, the F protein alone is sufficient to mediate viral penetration into host cells and subsequent syncytium formation during RSV infection (Feldman et al., 2000). As it can be seen in Figure 3 (right panel), **10d** was effective in reducing both syncytia area and number at a concentration of 50 μM. This evidence suggests that **10d** could interfere with the RSV F protein-mediated syncytium formation.

### Assessment of Antiviral Activity of 10d Against RSV Strain B1 cp-52

To confirm the results obtained from the syncytium reduction assay reported above, **10d** was further tested for activity in cells infected by RSV strain B1 cp-52. cp-52 is an RSV mutant variant containing a large deletion that ablates the synthesis of the SH and G glycoproteins. Nonetheless, cp-52 is infectious and replicates to high titer in tissue cultures (Karron et al., 1997). In the plaque-reduction assay, **10d** inhibited RSV cp-52 activity with 50% inhibitory concentration of 7 ± 1 μM, a value comparable to the one determined for the wild-type strain (EC_50_ = 2 μM, Table 1). In analogy, the two alternative potent compounds **10b** and **8d**, tested under the same conditions, gave comparable results (50% inhibitory concentration = 7 ± 1 μM and 10 ± 2 μM or **10b** and **8d**, respectively). These data support the hypothesis of the involvement of **10d** in fusion events via interaction with the RSV fusion F glycoprotein.

### Inhibition of RSV F Protein-Mediated Fusion Process by 10d

**Figure 4:**
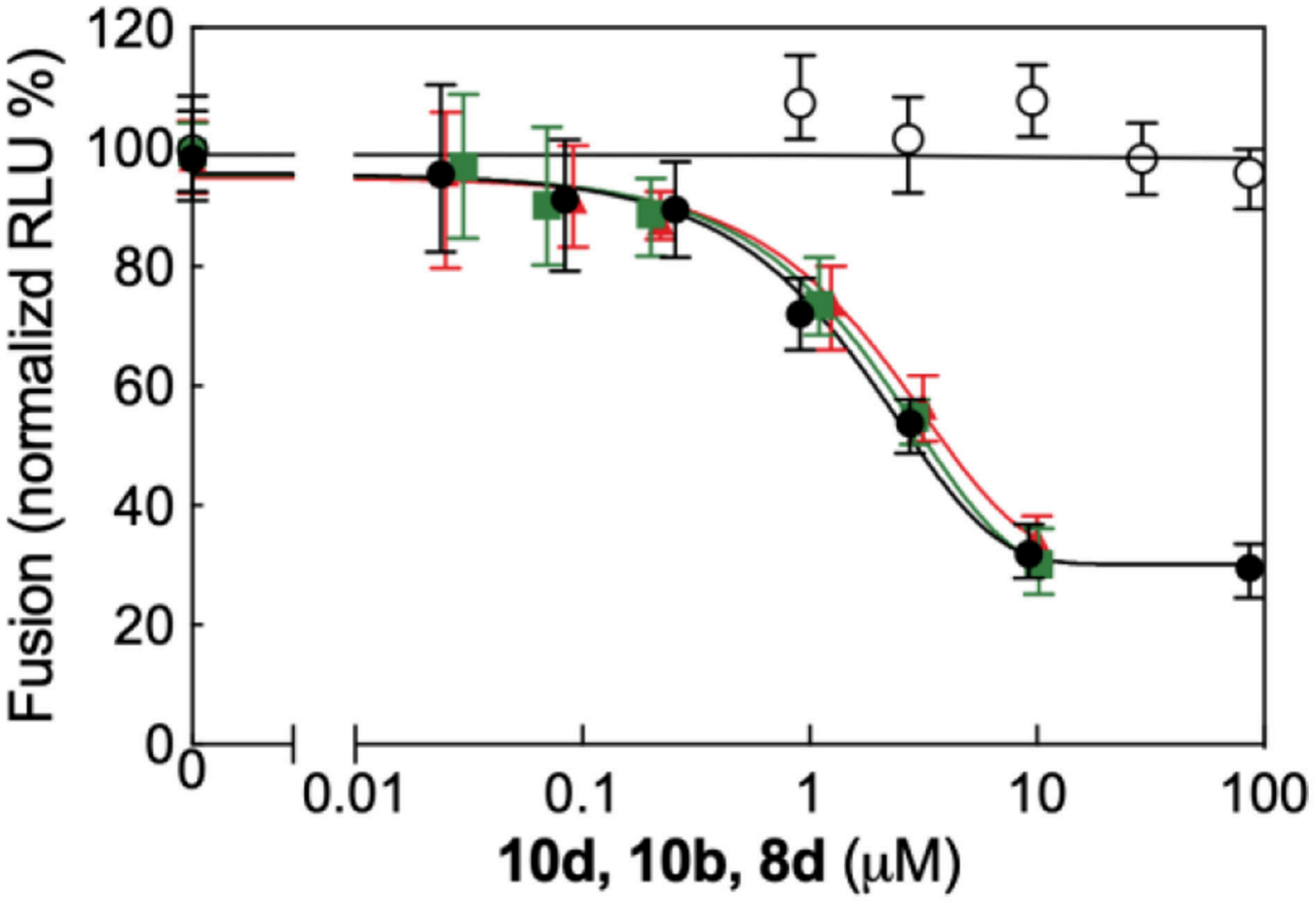
Quantitative dose-response cell-to-cell fusion assay using the DSP-chimeric reporter proteins and the ViviRenrenilla luciferase substrate in the presence of compounds **10d** (black filled symbols), **10b** (green filled symbols), and **8d** (red filled symbols). The MeV (Measles Virus) F and H glycoprotein expression constructs (open symbols) were included for selectivity control. Reported values are normalized for DMSO-treated samples and are expressed as the mean of three experiments ± standard deviation. The EC_50_ valuesfor the three compounds, obtained by 4-parameter variable slope regression fitting, are: 3.2 μM for **10d**, 3.9 μM for **10b**, and 4.5 μM for **8d**, respectively. Data for **10b** and **8d** were obtained under the same conditions employed for **10d** (see main text, Materials and Methods section).

All biological results discussed above support the inhibition of viral entry through involvement of fusion process as the possible mechanisms of action of compound **10d**. To investigate this aspect in more detail **10d** was tested in a plasmid-based reporter assay (Yan et al., 2014) that quantifies the bioactivity of viral entry (Nakane and Matsuda, 2015). According to this experiment (Figure 3, right panel), compound **10d** was able to specifically inhibit the RSV F protein-mediated membrane fusion process, with an EC_50_ value of 3.2 μM (90% confidence interval = 2.9–3.6). Repeating the same assay using the two alternative, potent compounds **8d** and **10b** yielded completely analogous results (Figure 4).

Taken together, all these data point to the interference with a protein F-mediated membrane merger as the underlying mechanism of anti-RSV activity of compound **10d**.

### *In silico* Interaction of 10d With the RSV F Glycoprotein

**Figure 5:**
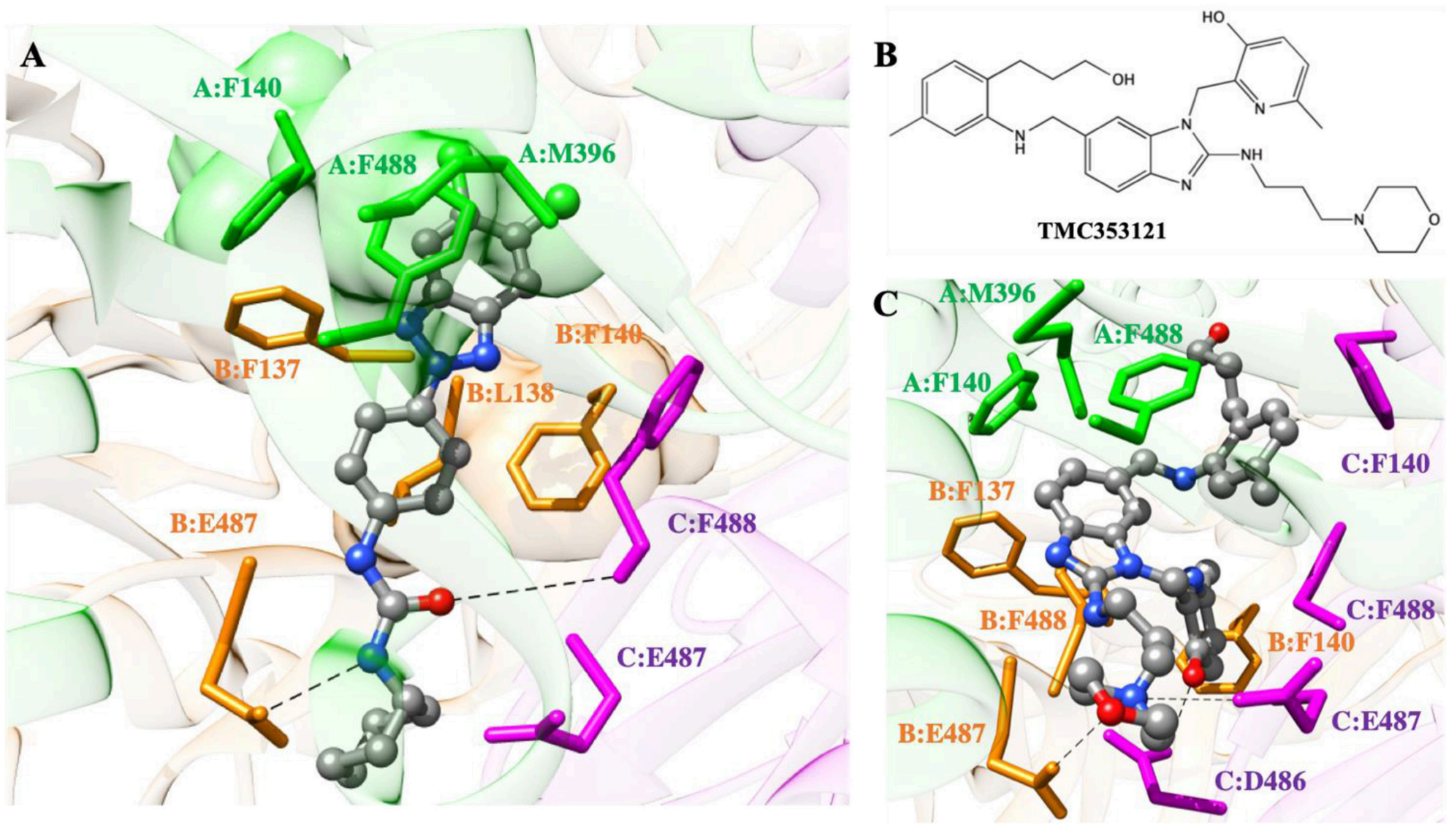
**(A)** Putative binding mode of compound **10d** into the 3-fold symmetric trimeric RSV F-protein in its pre-fusion state. The compound is shown as atom-colored balls-and-sticks (gray, C; blue, N; red, O; green, Cl). The three F protomers are represented as colored ribbons (light green, protomer A; light orange, protomer B; light purple, protomer C). The protein residues mainly involved in **10d** binding are evidenced and labeled. Hydrogen bonds are depicted as broken black lines. Hydrogen atoms, water molecules, ions and counterions are omitted for clarity. **(B)** Structure of the known RSV F-protein inhibitor TMC353121. **(C)** Details of TMC353121 in the binding pocket of the three-fold symmetric trimeric RSV F-protein in its pre-fusion state. Representations and colors as in **(A)**.

As the last step in the characterization of the antiviral activity of **10d**, its putative binding mode onto the RSV F-protein was predicted by molecular modeling (Figure 5A). According to the *in silico* experiment, **10d** could bind the three-fold symmetric trimeric protein in its pre-fusion state with a calculated IC_50_ of 8.2 μM (ΔG_bind_ = −7.22 ± 0.28 kcal/mol). The binding process was estimated to be enthalpy-driven (ΔH_bind_ = −21.37 ± 0.16 kcal/mol and -TΔS_bind_ = +14.15 ± 0.24 kcal/mol, respectively), in agreement with evidences from other RSV fusion inhibitors (Battles et al., 2016).

As seen in Figure 5A, the dichloro-substituted benzotriazole moiety is encased in a hydrophobic cavity lined by the side chains of F140, M396, and F488 (protomer A), whilst the phenyl ring of **10d** is engaged in π-π stacking with F140 of protomer B and other favorable hydrophobic interactions with the side chains of residues F137 and L138 of the same protomer. Moreover, two weak yet persistent hydrogen bonds are detected between **10d** and the protein. The first involves the oxygen atom of the ureidic group of **10d** and the E487-F488 backbone NH group of protomer C. The alternative H-bond is provided by the ureidic NH group of the compound and the side chain of E487 on protomer B.

According to the cell-based assay (Table 1), compounds **8d** and **10b** exhibited an antiviral activity comparable to that of **10d** (EC_50_ = 3 μM). Thus, we speculated that these two compounds could adopt a binding mode onto the trimeric RSV F-protein similar to that proposed for **10d**. To verify this hypothesis, the same computational approach was applied to the **8d**/ and **10b**/protein complexes (Figure S2). As expected, the replacement of the cyclohexyl ring of **10d** with a propyl chain in **10b** did not lead to any significant variation in the interaction spectrum described for **10d**: indeed, both fundamental hydrogen bonds and the nice molecular encasement in the protein hydrophobic cavities are still detected along the entire simulation trajectory (Figure S2A). Accordingly, the binding energetic profile is utterlt comparable to that calculated for the **10d**/protein complex with a ΔG_bind_ of −7.18 ± 0.31 kcal/mol (ΔH_bind_ = −21.28 ± 0.18 kcal/mol and -TΔS_bind_ = +14.10 ± 0.25 kcal/mol, IC_50_ = 8.7 μM). On the other hand, the replacement of the ureidic spacer with an amidic moiety in **8d** allows to perform only the hydrogen bond involving the compound oxygen atom (Figure S2B). Despite this, **8d** is provided with a good affinity against the RSV F-protein, with a calculated IC_50_ of 12.5 μM (ΔG_bind_ = −6.96 ± 0.29 kcal/mol). Although a slight decrease in the enthalpic contribution has been detected (ΔH_bind_ = −20.32 ± 0.16 kcal/mol), **8d** compensates with a less entropic penalty, justified from the presence of a more rigid 3,4,5-trimethoxybenzamide group in its structure.

With respect to current, highly potent inhibitors of RSV fusion process (e.g., TMC353121 or BMS433771) (Battles et al., 2016), **10d** is less potent since, being somewhat smaller in structure, it is not able to fully exploit the same plethora of stabilizing interactions that underlay the binding mode of e.g., TMC353121 (Figures 5B,C). Specifically, the hydrophobic pocket of protomer A (consisting of residues F140, M396, and F488) and the side chains of F137 and F488 of protomer B can properly accommodate the benzimidazole moiety of TMC353121 through favorable hydrophobic interactions. The morpholine group of the inhibitor optimizes protein binding by exploiting specific electrostatic interactions, i.e., the positively charged hydrogen atom performs a permanent ionic bridge with E487 of protomer C, while the oxygen atom is engaged in a stable dipole interaction with the side-chain of E487 (protomer B). Actually, the better binding performance of TMC353121 with respect to **10d** is related to the presence of two bulky substituents on its benzimidazole ring: a) the (3-hydroxypropyl)-5-methylphenyl moiety, which favorably interacts with the aromatic side chain of F140 of protomer C via a π-π stacking, and b) the 2-hydroxy-3-methylpiridin group, which not only performs positive hydrophobic interactions with F140 (protomer B) and F488 (protomer C) but is also engaged in a hydrogen bond with E487 (protomer C). The corresponding binding thermodynamics for the TMC353121/F-protein complex formation, i.e., ΔG_bind_ = −10.96 ± 0.31 kcal/mol, ΔH_bind_ = −26.57 ± 0.19 kcal/mol, and -TΔS_bind_ = +15.61 ± 0.25 kcal/mol), reflect the high affinity of this compound in binding the RSV F-protein, with a calculated IC_50_ of 19 nM, in very good agreement with the corresponding experimental value (IC_50_ = 8.7 nM, Battles et al., 2016).

In summary, preliminary computer-assisted drug design results reveal the possible binding mode of **10d** onto the RSV F-protein, justify at the molecular level the lower potency of this compound with respect to the nanomolar RSV F-protein inhibitor TMC353121, and suggest that further optimization of both the benzotriazole ring and the ureidic group substitutions of **10d** could lead to a second generation of more, potent RSV inhibitors. Efforts in this direction are currently ongoing in our laboratories.

## Conclusion

RSV is a widespread pathogen causing human lower respiratory-tract infections in people of all ages. Despite its long history, safe and effective cures for RSV remain elusive. In this work, a series of new 5,6-dichloro-phenylbenzotriazole derivatives were synthesized and tested for antiviral activity against RSV. All compounds were generally endowed with high activity (EC_50_ in the low micromolar range), low cytotoxicity (CC_50_ in the high micromolar range), and very high RSV selectivity in whole-virus cell-based assays. Among the entire class of compounds, **10d** was identified as a possible lead compound, with a potent anti-RSV activity (EC_50_ = 2 μM), high RSV selectivity, and an interesting safety profile against most continuous cell lines from different organs and species. As such, it was selected for further activity and mechanistic studies.

ELISA data revealed a dose-dependent reduction of viral antigen by **10d** at non-cytotoxic concentrations, whist a negligible virucidal effect of **10d** was found against RSV virions. These results indicated that the compound could not induce significant virion inactivation and that the plaque-reduction assay data were possibly due to the interference of **10d** with some step of the viral replication cycle.

Cell pretreatment with **10d** failed to prevent viral infection, and time of drug addition assays confirmed that **10d** effectively inhibited RSV activity in infected cells only when added during or immediately after infection. Both these results supported the hypothesis that **10d** could inhibit viral infection by hampering the binding and penetration processes of the virus into the host cells.

To discriminate between these two mechanisms, immunofluorescence assays were performed from which it clearly resulted that **10d** was not able to block viral binding to cells. Moreover, a dose-dependent reduction of syncytia area and number by **10d** led to the final speculation that the fusion of the viral envelop with the cell membrane, mediated by the RSV F glycoprotein, could be the molecular mechanism underlying the antiviral activity exerted by **10d**.

To confirm this hypothesis. the activity of **10d** was measured in cells infected with a RSV strain expressing only the surface F glycoprotein instead of the complete pool of F, G, and SH glycoproteins. An EC_50_ of 7 μM, comparable to the value measured for the wild-type virus (2 μM) indicated that the RSV F-glycoprotein could be the viral molecular target of **10d**. A subsequent plasmid-based reported assay confirmed that **10d** was able to specifically inhibit the RSV F protein-mediated membrane fusion process, with an EC_50_ value of 3.2 μM.

Finally, molecular simulations were used to predict the putative binding mode onto the RSV F glycoprotein in its pre-fusion state. All three protomers were found to be involved in binding **10d** via stabilizing hydrogen bonds, π-π and other hydrophobic interactions, ultimately resulting in an IC_50_ value of 8.2 μM. A comparison with the known and potent RSV F-protein inhibitor (TMC353121) highlighted the molecular level the lower potency of this compound

Given all the results presented and discussed above, it might be concluded that **10d** represents a promising and selective inhibitor of RSV which, in turn, could be considered as a good starting point for the development of second-generation effective candidate for early treatment of RSV infection.

## Author Contributions

AC and SPr conceived and designed the experiments. SPi, AC, PCo, and RI synthesized the compounds. GS, RL, SM, and PCa performed virus-related experiments. EL, SA, MF, and SPr performed the plasmid-based reporter assay and the *in silico* experiments. All authors analyzed data and contributed new reagents analytic tools. AC and SPr wrote the paper. All authors reviewed and approved the manuscript.

### Conflict of Interest Statement

The authors declare that the research was conducted in the absence of any commercial or financial relationships that could be construed as a potential conflict of interest.

## References

[B1] Battles M. B. Langedijk J. P. Furmanova-Hollenstein P. Chaiwatpongsakorn S. Costello H. M. Kwanten L.. (2016). Molecular mechanism of respiratory syncytial virus fusion inhibitors. Nat. Chem. Biol. 12, 87–93. 10.1038/nchembio.1982 26641933 PMC4731865

[B2] Briguglio I. Piras S. Corona P. Gavini E. Nieddu M. Boatto G. (2015). Benzotriazole: an overview on its versatile biological behaviour. Eur. J. Med. Chem. 97, 612–648. 10.1016/j.ejmech.2014.09.089 25293580 PMC7115563

[B3] Brindley M. A. Takeda M. Plattet P. Plemper R. K. (2012). Triggering the measles virus membrane fusion machinery. Proc. Natl. Acad. Sci. U.S.A. 109, 3018–3027. 10.1073/pnas.1210925109 23027974 PMC3497790

[B4] Budge P. J. Li Y. Beeler J. A. Graham B. S. (2004). RhoA-derived peptide dimers share mechanistic properties with other polyanionic inhibitors of respiratory syncytial virus (RSV), including disruption of viral attachment and dependence on RSV G. J. Virol. 78, 5015–5022. 10.1128/JVI.78.10.5015-5022.2004 15113882 PMC400344

[B5] Cardenas S. Auais A. Piedimonte G. (2005). Palivizumab in the prophylaxis of respiratory syncytial virus infection. Expert Rev. Anti-Infect. Ther. 3, 719–726. 10.1586/14787210.3.5.719 16207163

[B6] Carta A. Loriga G. Piras S. Paglietti G. Ferrone M. Fermeglia M. (2006). Synthesis and *in vitro* evaluation of the anti-viral activity of N-[4-(1H(2H) benzotriazol-1(2)-yl)phenyl]alkylcarboxamides. Med. Chem. 2, 577–589. 10.2174/1573406410602060577 17105439

[B7] Carta A. M. Loriga M. Piras S. Paglietti G. Ferrone M. Fermeglia M. (2007). Synthesis and anti-picornaviridae *in vitro* activity of a new class of helicase inhibitors the N,N'-bis[4-(1*H*(2*H*)-benzotriazol-1(2)-yl)fenyl]alkyldicarboxamides. Med. Chem. 3, 520–532. 10.2174/157340607782360308 18045201

[B8] Case D. A. Cerutti D. S. Cheatham T. E. III. Darden T. A. Duke R. E. Giese T. J. (2017). AMBER 2017. San Francisco, CA: University of California.

[B9] Falsey A. R. (2007). Respiratory syncytial virus infection in adults. Semin. Respir. Crit. Care Med. 28, 171–181. 10.1055/s-2007-976489 17458771

[B10] Falsey A. R. Hennessey P. A. Formica M. A. Cox C. Walsh E. E. (2005). Respiratory syncytial virus infection in elderly and high-risk adults. N. Engl. J. Med. 352, 1749–1759. 10.1056/NEJMoa043951 15858184

[B11] Feldman S. A. Audet S. Beeler J. A. (2000). The fusion glycoprotein of human respiratory syncytial virus facilitates virus attachment and infectivity via an interaction with cellular heparan sulfate. J. Virol. 74, 6442–6447. 10.1128/JVI.74.14.6442-6447.2000 10864656 PMC112152

[B12] Feltes T. F. Cabalka A. K. Meissner H. C. Piazza F. M. Carlin D. A. Top F. H. Jr.. (2003). Palivizumab prophylaxis reduces hospitalization due to respiratory syncytial virus in young children with hemodynamically significant congenital heart disease. J. Pediatr. 143, 532–540. 10.1067/S0022-3476(03)00454-2 14571236

[B13] Genini D. Brambilla L. Laurini E. Merulla J. Civenni G. Pandit S.. (2017). Mitochondrial dysfunction induced by a SH2 domain-targeting STAT3 inhibitor leads to metabolic synthetic lethality in cancer cells. Proc. Natl. Acad. Sci. U.S.A. 114, 4924–4933. 10.1073/pnas.1615730114 28584133 PMC5488915

[B14] Gibbons D. L. Pricl S. Posocco P. Laurini E. Fermeglia M. Sun H.. (2014). Molecular dynamics reveal BCR-ABL1 polymutants as a unique mechanism of resistance to PAN-BCR-ABL1 kinase inhibitor therapy. Proc. Natl. Acad. Sci. U.S.A. 111, 3550–3555. 10.1073/pnas.1321173111 24550512 PMC3948238

[B15] Heminway B. R. Yu Y. Tanaka Y. Perrine K. G. Gustafson E. Bernstein J. M.. (1994). Analysis of respiratory syncytial virus F, G, and SH proteins in cell fusion. Virology 200, 801–805. 10.1006/viro.1994.1245 8178462

[B16] Kahn J. S. Schnell M. J. Buonocore L. Rose J. K. (1999). Recombinant vesicular stomatitis virus expressing respiratory syncytial virus (RSV) glycoproteins: RSV fusion protein can mediate infection and cell fusion. Virology 254, 81–91. 10.1006/viro.1998.9535 9927576

[B17] Karron R. A. Buonagurio D. A. Georgiu A. F. Whitehead S. S. Adamus J. E. Clements-Mann M. L. (1997). Respiratory syncytial virus (RSV) SH and G proteins are not essential for viral replication *in vitro*: clinical evaluation and molecular characterization of a cold-passaged, attenuated RSV subgroup B mutant. Proc. Natl. Acad. Sci. U.S.A. 94, 13961–13966. 10.1073/pnas.94.25.13961 9391135 PMC28415

[B18] Kondo N. Miyauchi K. Matsuda Z. (2011). Monitoring viral-mediated membrane fusion using fluorescent reporter methods. Curr. Prot. Cell Biol. 26:Unit 26.29. 10.1002/0471143030.cb2609s50 21400700

[B19] Lee N. Qureshi S. T. (2013). Other viral pneumonias: coronavirus, respiratory syncytial virus, adenovirus, hantavirus. Crit. Care Clin. 29, 1045–1068. 10.1016/j.ccc.2013.07.003 24094390 PMC7126722

[B20] Maric I. Viaggi P. Caria P. Frau D. V. Degan P. Vanni R. (2011). Centrosomal and mitotic abnormalities in cell lines derived from papillary thyroid cancer harboring specific gene alterations. Mol. Cytogenet. 16:26, 1–8. 10.1186/1755-8166-4-26 PMC3248874 22087789

[B21] Massova I. Kollman P. A. (2000). Combined molecular mechanical and continuum solvent approach (MM-PBSA/GBSA) to predict ligand binding. Perspect. Drug Discov. 18, 113–135. 10.1023/A:1008763014207

[B22] Morris G. M. Huey R. Lindstrom W. Sanner M. F. Belew R. K. Goodsell D. S. (2016). Autodock4 and AutoDockTools4: automated docking with selective receptor flexiblity. J. Comput. Chem. 16, 2785–2791. 10.1002/jcc.21256 PMC2760638 19399780

[B23] Nair H. Nokes J. D. Gessner B. D. Dherani M. Madhi S. A. Singleton R. J.. (2010). Global burden of acute lower respiratory infections due to respiratory syncytial virus in young children: a systematic review and meta-analysis. Lancet 375, 1545–1555. 10.1016/S0140-6736(10)60206-1 20399493 PMC2864404

[B24] Nakane S. Matsuda Z. (2015). Cell Fusion, ed K. Pfannkuche (New York, NY: Humana Press), 229–236.

[B25] Noyola D. E. Zuviri-González A. Castro-García J. A. Ochoa-Zavala J. R. (2007). Impact of respiratory syncytial virus on hospital admissions in children younger than 3 years of age. J. Infect. 54, 180–184. 10.1016/j.jinf.2006.02.004 16580073

[B26] Ohmit S. E. Moler F. W. Monto A. S. Khan A. S. (1996). Ribavirin utilization and clinical effectiveness in children hospitalized with respiratory syncytial virus infection. J. Clin. Epidemiol. 49, 963–967. 10.1016/0895-4356(96)00137-0 8780603

[B27] Pauwels R. Balzarini J. Baba M. Snoeck R. Schols D. Herdewijn P.. (1988). Rapid and automated tetrazolium-based colorimetric assay for the detection of anti-HIV compounds. J. Virol. Methods. 20, 309–321. 10.1016/0166-0934(88)90134-6 2460479

[B28] Pierotti M. A. Tamborini E. Negri T. Pricl S. Pilotti S. (2011). Targeted therapy in GIST: *in silico* modeling for prediction of resistance. Nat. Rev. Clin. Oncol. 8, 161–170. 10.1038/nrclinonc.2011.3 21364689

[B29] Sanna G. Farci P. Busonera B. Murgia G. La Colla P. Giliberti G. (2015). Antiviral properties from plants of the Mediterranean flora. Nat. Prod. Res. 29, 2065–2070. 10.1080/14786419.2014.1003187 25613403

[B30] Sanna P. Carta A. Paglietti G. Zanetti S. Fadda G. (1992). 1,2,3-Triazolo[4,5-h]quinolines. III. Preparation and antimicrobial evaluation of 4-ethyl-4, 7.dihidro-1(2)-R-1(2)H-triazolo[4,5-h]quinolin-7-one-6-carboxylic acids. Il Farmaco. 47, 1001–1019. 1332728

[B31] Snell N. J. (2001). Ribavirin-current status of a broad spectrum antiviral agent. Expert. Opin. Pharmacother. 2, 1317–1324. 10.1517/14656566.2.8.1317 11585000

[B32] Techaarpornkul S. Barretto N. Peeples M. E. (2001). Functional analysis of recombinant respiratory syncytial virus deletion mutants lacking the small hydrophobic and/or attachment glycoprotein gene. J. Virol. 75, 6825–6834. 10.1128/JVI.75.15.6825-6834.2001 11435561 PMC114409

[B33] Ventre K. Randolph A. G. (2007). Ribavirin for respiratory syncytial virus infection of the lower respiratory tract in infants and young children. Cochrane Data. Syst. Rev. 24:CD000181 10.1002/14651858.CD000181.pub3 17253446

[B34] Wang E. E. Law B. J. Stephens D. (1995). Pediatric Investigators Collaborative Network on Infections in Canada (PICNIC) prospective study of risk factors and outcomes in patients hospitalized with respiratory syncytial viral lower respiratory tract infection. J. Pediatr. 126, 212–219. 10.1016/S0022-3476(95)70547-3 7844667

[B35] Yan D. Lee S. Thakkar V. D. Moore M. L. Plemper R. K. (2014). Cross-resistance mechanism of respiratory syncytial virus against structurally diverse entry inhibitors. Proc. Natl. Acad. Sci. U.S.A. 11, 3441–3449. 10.1073/pnas.1405198111 PMC4143008 25092342

