# Supplementary material for: Dichloro-Phenyl-Benzotriazoles: A New Selective Class of Human Respiratory Syncytial Virus Entry Inhibitors
Source: Front Chem. 2019 Apr 16;7:247. doi: 10.3389/fchem.2019.00247 (PMC6476926; doi:10.3389/fchem.2019.00247)
Supplement: Supplementary file 1 [file Data_Sheet_1.PDF]

Dichloro-phenyl-benzotriazoles: a new selective class of Human Respiratory Syncytial virus entry inhibitors

Sandra Piras[a], Giuseppina Sanna[b], Antonio Carta[a,*], Paola Corona[a], Roberta Ibba[a], Roberta Loddo[b,*], Silvia Madeddu[b], Paola Caria[b], SuzanaAulic[c], Erik Laurini[c,], Maurizio Fermeglia[c], Sabrina Pricl[c].

[a] Department of Chemistry and Pharmacy, University of Sassari, IT, Italy.

[b] Department of Biomedical Sciences, University of Cagliari, IT, Italy.

[c] Molecular Simulation Engineering (MOSE) Laboratory, University of Trieste, IT, Italy.

TABLE S1. Activity of 5,6-dichloro-1-phenyl-benzotriazole amides(**5a-d** and **7a-h**) against viruses representative of positive-sense, single-stranded RNAs (ssRNA+): i) Retroviridae: HIV-1; ii) Flaviviridae: YFV and BVDV; iii) Picornaviridae: CV-B5 and Sb-1. Viruses representative of negative-sense, single-stranded RNAs (ssRNA-); i) Rhabdoviridae: VSV. Virus representative of double-stranded RNAs (dsRNA): Reoviridae: Reo-1. DNA virus representatives: i) Poxviridae: VV; ii) Herpesviridae: HSV-1. Efavirenz, 2’-C-methyl-guanosine, and Pleconarilwere used as reference inhibitors.Data represent mean values ± SD for three independent determinations. For values where SD is not shown, variation among duplicate samples was less than 15%.Efavirenz (EFV), 2’-C-methyl-guanosine (2MG), and Pleconaril (PCL) were used as reference inhibitors.

Cpd	MT-4 cells	$HIV\text{-}1_{IIIB}$	MDBK cells	BVDV	BHK cells	YFV	Reo-1	Vero-76 cells	CV-B5	Sb-1, VSV, VV, HSV-1
	CC_{50}[a]	EC_{50}[b]	CC_{50}[c]	EC_{50}[d]	CC_{50}[e]	EC_{50}[f]	EC_{50}[g]	CC_{50}[h]	EC_{50}[i]	EC_{50}[j]
**1a**	>100	>100	>100	**75**	>100	>100	>100	>100	85	>100
**5a**	35	>35	43	>43	53	>53	>53	30	**17**	>30
**5b**	28	>28	>100	>100	54	>54	>54	30	>30	>30
**5c**	60	>60	>100	>100	>100	>100	>100	10	**9**	>100

**5d**	35	>35	14	>14	16	>16	>16	20	>20	>20
**7a**	>100	>100	>100	>100	44	>44	>44	>100	>100	>100
**7b**	>100	>100	>100	>100	>100	>100	>100	>100	>100	>100
**7c**	33	>33	>100	>100	>100	>100	>100	>100	>100	>100
**7d**	77	>77	>100	>100	>100	**78**	>100	90	>90	>90
**7e**	>100	>100	>100	>100	96	>96	>96	>100	>100	>100
**7f**	>100	>100	>100	>100	84	>84	>84	>100	>100	>100
**7g**	>100	>100	>100	>100	>100	>100	>100	9	>90	>90
**7h**	>100	>100	>100	>100	>100	>100	>100	>100	>100	>100
**Ref Cpd**										
**EFV**	40	0.002 ± 0.0002								
**2MG**			>100	1.1 ± 0.1	>100	1.9 ± 0.1	0.7 ± 0.2			
**PCL**								>100	0.005 ± 0.001	

[a]Compound concentration (µM) required to reduce the proliferation of mock-infected MT-4 cells by 50%, as determined by the MTT method. [b]Compound concentration (µM) required to achieve 50% protection of MT-4 cells from HIV-1 induced cytopathogenicity, as determined by the MTT method. [c]Compound concentration (µM) required to reduce the viability of mock-infected MDBK cells by 50%, as determined by the MTT method. [d]Compound concentration (µM) required to achieve 50% protection of MDBK cells from BVDV-induced cytopathogenicity, as determined by the MTT method. [e]Compound concentration (µM) required to reduce the viability of mock-infected BHK cells by 50%, as determined by the MTT method. [f]Compound concentration (µM required to achieve 50% protection of BHK cells from YFV-induced cytopathogenicity, as determined by the MTT method. [g]Compound concentration (µM) required to achieve 50% protection of BHK cells from Reo-1-induced cytopathogenicity, as determined by the MTT method. [h]Compound concentration (µM) required to reduce the viability of mock-infected VERO-76 cells by 50%. as determined by the MTT method. [i]Compound concentration (µM) required to reduce the plaque number of CV-B5 by 50% in VERO-76 monolayers. [j]Compound concentration (µM) required to reduce the plaque number of Sb-1, VSV, VV and HSV-1 by 50% in VERO-76 monolayers.

TABLE S2. Activity of 5,6-dichloro-2-phenyl-benzotriazole amides(**6a-h** and **8a-h**), and

5,6-dichloro-2-phenyl-benzotriazole urees**(10a-k)** against viruses representative of positive-sense, single-stranded RNAs (ssRNA+): i) Retroviridae: HIV-1; ii) Flaviviridae: YFV and BVDV; iii) Picornaviridae: CV-B5 and Sb-1. Viruses representative of negative-sense, single-stranded RNAs (ssRNA-); i) Rhabdoviridae: VSV. Virus representative of double-stranded RNAs (dsRNA): Reoviridae: Reo-1. DNA virus representatives: i) Poxviridae: VV; ii) Herpesviridae: HSV-1. For values where SD is not shown, variation among duplicate samples was less than 15%.Efavirenz (EFV), 2'-C-methyl-guanosine (2MG), and Pleconaril (PCL) were used as reference inhibitors.

**Cpd**	**MT-4 cells**	**HIV-1$_{IIIB}$**	**MDBK cells**	**BVDV**	**BHK cells**	**YFV**	**Reo-1**	**Vero-76 cells**	**CV-B5**	**Sb-1, VSV, VV, HSV-1**
	CC_{50}[a]	EC_{50}[b]	CC_{50}[c]	EC_{50}[d]	CC_{50}[e]	EC_{50}[f]	EC_{50}[g]	CC_{50}[h]	EC_{50}[i]	EC_{50}[j]
**1b**	52	>52	≥100	20	>100	>100	>100	>100	>100	>100
**6a**	>100	>100	>100	>100	>100	>100	>100	>100	>100	>100
**6b**	>100	>100	>100	>100	>100	>100	>100	>100	>100	>100
**6c**	>100	>100	>100	>100	>100	>100	>100	>100	>100	>100
**6d**	33	>33	100	>100	72	>72	>100	>100	>100	>100
**6e**	15	>15	72	>72	26	>26	>26	>100	**61**	>100
**6f**	24	>24	84	>84	62	>62	>26	>100	**33**	>100
**8a**	>100	>100	>100	>100	>100	>100	>100	>100	>100	>100
**8b**	>100	>100	>100	>100	>100	**73**	>100	>100	>100	>100
**8c**	>100	>100	>100	>100	>100	>100	>100	>100	**14**	>100
**8d**	63	>63	>100	**35**	35	>35	>35	80	>80	>80
**8e**	>100	>100	>100	**4**	68	>68	>68	>100	>100	>100
**8f**	>100	>100	>100	**60**	>100	>100	>100	80	>100	>100
**8g**	>100	>100	>100	**28**	>100	>100	>100	>100	>100	>100

**8h**	>100	>100	>100	**11**	>100	>100	>100	>100	>100	>100
**10a**	>100	>100	>100	>100	>100	>100	>100	>100	>100	>100
**10b**	>100	>100	78	>78	40	>40	>40	30	>30	>30
**10c**	>100	>100	>100	>100	>100	>100	>100	90	>95	>95
**10d**	>100	>100	>100	>100	71	>71	>71	90	>90	>90
**10e**	>100	>100	>100	>100	>100	>100	>100	>100	>100	>100
**10f**	>100	>100	>100	>100	>100	>100	>100	>100	>100	>100
**10g**	>100	>100	>100	>100	>100	>100	>100	>100	>100	>100
**10h**	>100	>100	>100	>100	>100	>100	>100	>100	>100	>100
**10i**	>100	>100	>100	>100	>100	>100	>100	>100	>100	>100
**10j**	>100	>100	>100	>100	>100	>100	>100	>100	>100	>100
**10k**	>100	>100	>100	>100	>100	>100	>100	>100	>100	>100
**Ref Cpd**										
**EFV**	40	0.002 ± 0.0002								
**2MG**			>100	1.1 ± 0.1	>100	1.9 ± 0.1	0.7 ± 0.2			
**PCL**								>100	0.005 ± 0.001	

[a]Compound concentration (μM) required to reduce the proliferation of mock-infected MT-4 cells by 50%, as determined by the MTT method. [b]Compound concentration (μM) required to achieve 50% protection of MT-4 cells from HIV-1 induced cytopathogenicity, as determined by the MTT method. [c]Compound concentration (μM) required to reduce the viability of mock-infected MDBK cells by 50%, as determined by the MTT method. [d]Compound concentration (μM) required to achieve 50% protection of MDBK cells from BVDV-induced cytopathogenicity, as determined by the MTT method. [e]Compound concentration (μM) required to reduce the viability of mock-infected BHK cells by 50%, as determined by the MTT method. [f]Compound concentration (μM   required to achieve 50% protection of BHK cells from YFV-induced cytopathogenicity, as determined by the MTT method. [g]Compound concentration (μM) required to achieve 50% protection of BHK cells from Reo-1-induced

cytopathogenicity, as determined by the MTT method. [h]Compound concentration (μM) required to reduce the viability of mock-infected VERO-76 cells by 50%. as determined by the MTT method. [i]Compound concentration (μM) required to reduce the plaque number of CV-B5 by 50% in VERO-76 monolayers. [j]Compound concentration (μM) required to reduce the plaque number of Sb-1, VSV, VV and HSV-1 by 50% in VERO-76 monolayers.

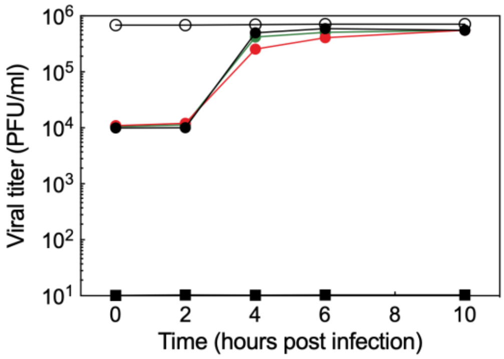

Figure S1. Inhibition of RSV (m.o.i = 1) by addition of 20 μM of compound **10d** (black filled circles), **10b** (green filled circles), and **8d** (red filled circles) at different times. Data for untreated virus (open circles) and for addition of 6-azauridine (filled squares) are also shown for comparison. Data represent mean values from two independent determinations; variation among duplicate samples was less than 15%. Data for **10b** and **8d** were obtained under the same conditions employed for **10d** (see main text, Materials and Methods section).

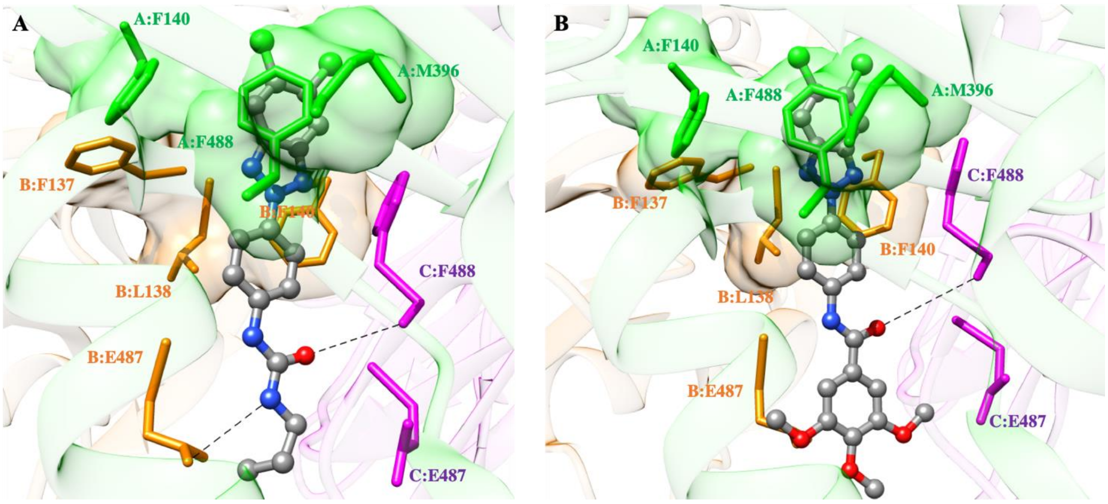

Figure S2. Putative binding mode of compound **10b** (A) and **8d** (B) into the three-fold symmetric trimeric RSV F-protein in its pre-fusion state. The compounds are shown as atom-colored balls-and-sticks (gray, C; blue, N; red, O; green, Cl). The three F protomers are represented as colored ribbons (green, protomer A; orange, protomer B; purple, protomer C). The protein residues mainly involved in **10d** binding are evidenced and labeled. Hydrogen bonds are depicted as broken black lines. Hydrogen atoms, water molecules, ions and counterions are omitted for clarity.
