# Supplementary material for: Dichloro-Phenyl-Benzotriazoles: A New Selective Class of Human Respiratory Syncytial Virus Entry Inhibitors
Source: Front Chem. 2019 Apr 16;7:247. doi: 10.3389/fchem.2019.00247 (PMC6476926; doi:10.3389/fchem.2019.00247)
Supplement: Supplementary file 2 [file Data_Sheet_2.PDF]

# Dichloro-phenyl-benzotriazoles: a new selective class of Human Respiratory Syncytial virus entry inhibitors


Sandra Piras[a], Giuseppina Sanna[b], Antonio Carta[a,*], Paola Corona[a], Roberta Ibba[a], Roberta Loddo[b,*], Silvia Madeddu[b], Paola Caria[b], SuzanaAulic[c], Erik Laurini[c,], Maurizio Fermeglia[c], Sabrina Pricl[c].

[a] Department of Chemistry and Pharmacy, University of Sassari, IT, Italy.

[b] Department of Biomedical Sciences, University of Cagliari, IT, Italy.

[c] Molecular Simulation Engineering (MOSE) Laboratory, University of Trieste, IT, Italy.


**TABLE S1.** Activity of 5,6-dichloro-1-phenyl-benzotriazole amides(**5a-d** and **7a-h**) against viruses representative of positive-sense, single-stranded RNAs (ssRNA+): i) Retroviridae: HIV-1; ii) Flaviviridae: YFV and BVDV; iii) Picornaviridae: CV-B5 and Sb-1. Viruses representative of negative-sense, single-stranded RNAs (ssRNA-); i) Rhabdoviridae: VSV. Virus representative of double-stranded RNAs (dsRNA): Reoviridae: Reo-1. DNA virus representatives: i) Poxviridae: VV; ii) Herpesviridae: HSV-1. Efavirenz, 2’-C-methyl-guanosine, and Pleconarilwere used as reference inhibitors.Data represent mean values ± SD for three independent determinations. For values where SD is not shown, variation among duplicate samples was less than 15%.Efavirenz (EFV), 2’-C-methyl-guanosine (2MG), and Pleconaril (PCL) were used as reference inhibitors.

| **Cpd** | **MT-4 cells** | **HIV-1$_{IIIB}$** | **MDBK cells** | **BVDV** | **BHK cells** | **YFV** | **Reo-1** | **Vero-76 cells** | **CV-B5** | **Sb-1, VSV, VV, HSV-1** |
|---|---|---|---|---|---|---|---|---|---|---|
| | $CC_{50}$[a] | $EC_{50}$[b] | $CC_{50}$[c] | $EC_{50}$[d] | $CC_{50}$[e] | $EC_{50}$[f] | $EC_{50}$[g] | $CC_{50}$[h] | $EC_{50}$[i] | $EC_{50}$[j] |
| **1a** | >100 | >100 | >100 | **75** | >100 | >100 | >100 | >100 | 85 | >100 |
| **5a** | 35 | >35 | 43 | >43 | 53 | >53 | >53 | 30 | **17** | >30 |
| **5b** | 28 | >28 | >100 | >100 | 54 | >54 | >54 | 30 | >30 | >30 |

| | | | | | | | | | | |
|---|---|---|---|---|---|---|---|---|---|---|
| **5c** | 60 | >60 | >100 | >100 | >100 | >100 | >100 | 10 | **9** | >100 |
| **5d** | 35 | >35 | 14 | >14 | 16 | >16 | >16 | 20 | >20 | >20 |
| **7a** | >100 | >100 | >100 | >100 | 44 | >44 | >44 | >100 | >100 | >100 |
| **7b** | >100 | >100 | >100 | >100 | >100 | >100 | >100 | >100 | >100 | >100 |
| **7c** | 33 | >33 | >100 | >100 | >100 | >100 | >100 | >100 | >100 | >100 |
| **7d** | 77 | >77 | >100 | >100 | >100 | **78** | >100 | 90 | >90 | >90 |
| **7e** | >100 | >100 | >100 | >100 | 96 | >96 | >96 | >100 | >100 | >100 |
| **7f** | >100 | >100 | >100 | >100 | 84 | >84 | >84 | >100 | >100 | >100 |
| **7g** | >100 | >100 | >100 | >100 | >100 | >100 | >100 | 9 | >90 | >90 |
| **7h** | >100 | >100 | >100 | >100 | >100 | >100 | >100 | >100 | >100 | >100 |
| **Ref Cpd** | | | | | | | | | | |
| **EFV** | 40 | 0.002 ± 0.0002 | | | | | | | | |
| **2MG** | | | >100 | 1.1 ± 0.1 | >100 | 1.9 ± 0.1 | 0.7 ± 0.2 | | | |
| **PCL** | | | | | | | | >100 | 0.005 ± 0.001 | |

[a]Compound concentration (μM) required to reduce the proliferation of mock-infected MT-4 cells by 50%, as determined by the MTT method. [b]Compound concentration (μM) required to achieve 50% protection of MT-4 cells from HIV-1 induced cytopathogenicity, as determined by the MTT method. [c]Compound concentration (μM) required to reduce the viability of mock-infected MDBK cells by 50%, as determined by the MTT method. [d]Compound concentration (μM) required to achieve 50% protection of MDBK cells from BVDV-induced cytopathogenicity, as determined by the MTT method. [e]Compound concentration (μM) required to reduce the viability of mock-infected BHK cells by 50%, as determined by the MTT method. [f]Compound concentration (μM required to achieve 50% protection of BHK cells from YFV-induced cytopathogenicity, as determined by the MTT method. [g]Compound concentration (μM) required to achieve 50% protection of BHK cells from Reo-1-induced cytopathogenicity, as determined by the MTT method. [h]Compound concentration (μM) required to reduce the viability of mock-infected VERO-76 cells by 50%. as determined by the MTT method. [i]Compound concentration (μM) required to reduce the plaque number of CV-B5 by 50% in VERO-76 monolayers. [j]Compound concentration (μM) required to reduce the plaque number of Sb-1, VSV, VV and HSV-1 by 50% in VERO-76 monolayers.

**TABLE S2.** Activity of 5,6-dichloro-2-phenyl-benzotriazole amides(**6a-h** and **8a-h**), and 5,6-dichloro-2-phenyl-benzotriazole urees**(10a-k)** against viruses representative of positive-sense, single-stranded RNAs (ssRNA+): i) Retroviridae: HIV-1; ii) Flaviviridae: YFV and BVDV; iii) Picornaviridae: CV-B5 and Sb-1. Viruses representative of negative-sense, single-stranded RNAs (ssRNA-); i) Rhabdoviridae: VSV. Virus representative of double-stranded RNAs (dsRNA): Reoviridae: Reo-1. DNA virus representatives: i) Poxviridae: VV; ii) Herpesviridae: HSV-1. For values where SD is not shown, variation among duplicate samples was less than 15%.Efavirenz (EFV), 2’-C-methyl-guanosine (2MG), and Pleconaril (PCL) were used as reference inhibitors.

| **Cpd** | **MT-4 cells** | **HIV-1$_{IIIB}$** | **MDBK cells** | **BVDV** | **BHK cells** | **YFV** | **Reo-1** | **Vero-76 cells** | **CV-B5** | **Sb-1, VSV, VV, HSV-1** |
|---|---|---|---|---|---|---|---|---|---|---|
| | $CC_{50}$[a] | $EC_{50}$[b] | $CC_{50}$[c] | $EC_{50}$[d] | $CC_{50}$[e] | $EC_{50}$[f] | $EC_{50}$[g] | $CC_{50}$[h] | $EC_{50}$[i] | $EC_{50}$[j] |
| **1b** | 52 | >52 | ≥100 | 20 | >100 | >100 | >100 | >100 | >100 | >100 |
| **6a** | >100 | >100 | >100 | >100 | >100 | >100 | >100 | >100 | >100 | >100 |
| **6b** | >100 | >100 | >100 | >100 | >100 | >100 | >100 | >100 | >100 | >100 |
| **6c** | >100 | >100 | >100 | >100 | >100 | >100 | >100 | >100 | >100 | >100 |
| **6d** | 33 | >33 | 100 | >100 | 72 | >72 | >100 | >100 | >100 | >100 |
| **6e** | 15 | >15 | 72 | >72 | 26 | >26 | >26 | >100 | **61** | >100 |
| **6f** | 24 | >24 | 84 | >84 | 62 | >62 | >26 | >100 | **33** | >100 |
| **8a** | >100 | >100 | >100 | >100 | >100 | >100 | >100 | >100 | >100 | >100 |
| **8b** | >100 | >100 | >100 | >100 | >100 | **73** | >100 | >100 | >100 | >100 |
| **8c** | >100 | >100 | >100 | >100 | >100 | >100 | >100 | >100 | **14** | >100 |
| **8d** | 63 | >63 | >100 | **35** | 35 | >35 | >35 | 80 | >80 | >80 |
| **8e** | >100 | >100 | >100 | **4** | 68 | >68 | >68 | >100 | >100 | >100 |
| **8f** | >100 | >100 | >100 | **60** | >100 | >100 | >100 | 80 | >100 | >100 |

| | | | | | | | | | | |
|---|---|---|---|---|---|---|---|---|---|---|
| **8g** | >100 | >100 | >100 | **28** | >100 | >100 | >100 | >100 | >100 | >100 |
| **8h** | >100 | >100 | >100 | **11** | >100 | >100 | >100 | >100 | >100 | >100 |
| **10a** | >100 | >100 | >100 | >100 | >100 | >100 | >100 | >100 | >100 | >100 |
| **10b** | >100 | >100 | 78 | >78 | 40 | >40 | >40 | 30 | >30 | >30 |
| **10c** | >100 | >100 | >100 | >100 | >100 | >100 | >100 | 90 | >95 | >95 |
| **10d** | >100 | >100 | >100 | >100 | 71 | >71 | >71 | 90 | >90 | >90 |
| **10e** | >100 | >100 | >100 | >100 | >100 | >100 | >100 | >100 | >100 | >100 |
| **10f** | >100 | >100 | >100 | >100 | >100 | >100 | >100 | >100 | >100 | >100 |
| **10g** | >100 | >100 | >100 | >100 | >100 | >100 | >100 | >100 | >100 | >100 |
| **10h** | >100 | >100 | >100 | >100 | >100 | >100 | >100 | >100 | >100 | >100 |
| **10i** | >100 | >100 | >100 | >100 | >100 | >100 | >100 | >100 | >100 | >100 |
| **10j** | >100 | >100 | >100 | >100 | >100 | >100 | >100 | >100 | >100 | >100 |
| **10k** | >100 | >100 | >100 | >100 | >100 | >100 | >100 | >100 | >100 | >100 |
| **Ref Cpd** | | | | | | | | | | |
| **EFV** | 40 | 0.002 ± 0.0002 | | | | | | | | |
| **2MG** | | | >100 | 1.1 ± 0.1 | >100 | 1.9 ± 0.1 | 0.7 ± 0.2 | | | |
| **PCL** | | | | | | | | >100 | 0.005 ± 0.001 | |

[a]Compound concentration (μM) required to reduce the proliferation of mock-infected MT-4 cells by 50%, as determined by the MTT method. [b]Compound concentration (μM) required to achieve 50% protection of MT-4 cells from HIV-1 induced cytopathogenicity, as determined by the MTT method. [c]Compound concentration (μM) required to reduce the viability of mock-infected MDBK cells by 50%, as determined by the MTT method. [d]Compound concentration (μM) required to achieve 50% protection of MDBK cells from BVDV-induced cytopathogenicity, as determined by the MTT method. [e]Compound concentration (μM) required to reduce the viability of mock-infected BHK cells by 50%, as determined by the MTT method. [f]Compound

concentration (μM required to achieve 50% protection of BHK cells from YFV-induced cytopathogenicity, as determined by the MTT method. [g]Compound concentration (μM) required to achieve 50% protection of BHK cells from Reo-1-induced cytopathogenicity, as determined by the MTT method. [h]Compound concentration (μM) required to reduce the viability of mock-infected VERO-76 cells by 50%. as determined by the MTT method. [i]Compound concentration (μM) required to reduce the plaque number of CV-B5 by 50% in VERO-76 monolayers. [j]Compound concentration (μM) required to reduce the plaque number of Sb-1, VSV, VV and HSV-1 by 50% in VERO-76 monolayers.

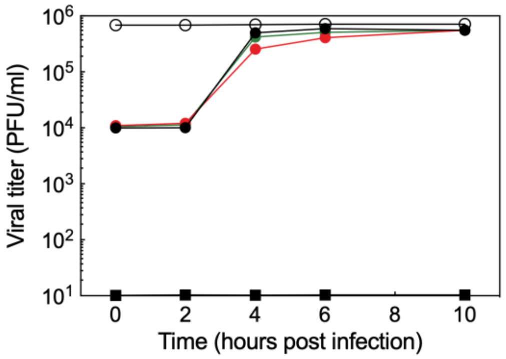


**Figure S1.**Inhibition of RSV (m.o.i = 1) by addition of 20 μM of compound **10d** (black filled circles), **10b** (green filled circles), and **8d** (red filled circles) at different times. Data for untreated virus (open circles) and for addition of 6-azauridine (filled squares) are also shown for comparison. Data represent mean values from two independent determinations; variation among duplicate samples was less than 15%. Data for **10b** and **8d** were obtained under the same conditions employed for **10d** (see main text, Materials and Methods section).

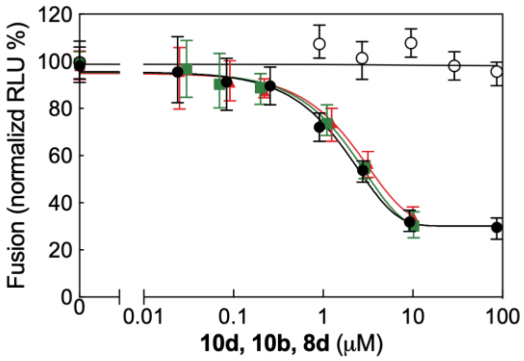


**Figure S2.**Quantitative dose-response cell-to-cell fusion assay using the DSP-chimeric reporter proteins and the ViviRenrenilla luciferase substrate in the presence of compounds**10d** (black filled symbols), **10b** (green filled symbols), and **8d** (red filled symbols). The MeV (Measles Virus) F and H glycoprotein expression constructs (open symbols) were included for selectivity control. Reported values are normalized for DMSO-treated samples and are expressed as the mean of three experiments ± standard deviation. The $EC_{50}$ valuesfor the three compounds, obtained by 4-parameter variable slope regression fitting, are: 3.2 μM for **10d**, 3.9 μM for **10b**, and 4.5 μM for **8d**, respectively. Data for **10b** and **8d** were obtained under the same conditions employed for **10d** (see main text, Materials and Methods section).
